# Grounding mathematics in an integrated conceptual structure, part II: intervention study demonstrating robust learning and retention through a grounded curriculum

**DOI:** 10.3389/fpsyg.2025.1507674

**Published:** 2026-01-02

**Authors:** Kevin W. Mickey, Laura W. Kreisel, Su Su, James L. McClelland

**Affiliations:** Department of Psychology, Stanford University, Stanford, CA, United States

**Keywords:** visuospatial representation, learning trigonometry, conceptual grounding, generalization, transfer learning, mathematical cognition

## Abstract

Mathematical reasoning systems are often treated as systems for manipulating formal expressions according to structure sensitive rules. However, these expressions typically reference objects, properties, and relationships in a target domain in which they have meaning. One case in point is precalculus trigonometry, a crucial part of high-school preparation for university level mathematics. In work reported in Part I of this 2-part publication, we found evidence that reliance on the unit circle, a visuospatial structure that provides meaning for formal expressions in trigonometry, provides an integrated conceptual framework that supports successful mastery of foundational trigonometric relationships that are often very difficult for students to learn. Importantly, however, although coverage of the unit circle is standard in classrooms and textbooks, many students fail to rely on it and so fail to benefit from the conceptual model it provides. Here, we consider some of the reasons why mastery of this system may be challenging and some of the pitfalls in the ways these relationships are often taught. We then describe a set of principles we used to guide the development of a tutorial curriculum aimed at addressing these challenges and pitfalls. After refinement, our curriculum was successful in allowing many high-school and community college students to learn to solve trigonometric identity problems students often fail to master in typical classroom settings and to retain what they had learned over a 2–3 week delay. Our findings demonstrate the value of developing structured, conceptually grounded, and research-motivated teaching materials to allow students to gain mastery of mathematical systems they might otherwise fail to learn.

## 1 Introduction

The ability to learn mathematics is becoming increasingly important, given the centrality of mathematics in scientific and technical disciplines, at a time when these disciplines are becoming more and more complex. Many countries, however, including the USA, face daunting challenges in educating students to master mathematical subjects (Fleischman et al., 2010). There are likely many factors that contribute to successful educational outcomes in mathematics. Here, we focus on the framing of what it means to acquire a basic understanding of a domain of mathematics, identification of barriers to acquiring such an understanding, and the development of practices that may contribute to successful outcomes.

In the first article in this two-part series (Mickey and McClelland, 2025), we explored the mathematical domain of precalculus trigonometry, presenting arguments and evidence for the idea that an important part of mastering a branch of mathematics lies in forming an integrated conceptual structure within which the mathematical expressions used in the domain have meaning. In the present article, we consider reasons why acquiring such an understanding in the trigonometry domain can be challenging for students, and we consider pitfalls in classroom instruction and textbooks that may limit students' success. Next, we describe principles and findings from the cognitive and educational sciences that guided our development of a multi-lesson curriculum aimed at helping students acquire an integrative conceptual structure for understanding trigonometric relationships. We then report a controlled intervention study in which we found that the resulting curriculum was successful in helping many students acquire an understanding of trigonometric relationships that are often challenging for students to learn. Though our work focuses on a specific domain of mathematics, we argue that similar considerations apply in many other mathematical domains. Thus, we suggest that our findings are of broad relevance, and we hold out the hope that future work will extend the approach and integrate its principles more fully into mathematics education in a wide range of mathematical and scientific domains.

### 1.1 The concept of an integrated conceptual structure

Our notion of an integrated conceptual structure builds on the work of Robbie Case (Case et al., 1996; Moss and Case, 1999) who used the phrase *central conceptual structure*, illustrating what he meant by this in work focused on his conceptualization of the mental number line. Case saw the number line as a highly structured representation system, rather than as a simple monotonic continuum as proposed by others (e.g., Dehaene, 1997). Case viewed the number line as a visuo-spatial structure that is closely coordinated with the notational scheme we use for representing numbers symbolically. Specifically, in Case et al. (1996), Case and his colleagues considered how the place-value system that we use to represent the numbers from 0 to 100 maps onto a physical number line with 0 at its left end and 100 on the right, with the line represented as a hierarchical structure divided into 10 decades, each of which is further divided into 10 units, and with milestones at significant points such as the middle of the line (corresponding to 50) and the quartile boundaries (25 and 75) along the line (Barth and Paladino, 2011). In relation to this structure, a number like 37 is understood to specify a particular point along the line, with the leftmost digit indicating which decade the point lies within and the next digit to the right indicating where the point falls within this decade. In Mickey and McClelland (2025), we introduced the phrase *integrated conceptual structure* instead of Case's central conceptual structure to emphasize how this system integrates both symbolic and visuospatial elements. In Moss and Case (1999) the authors extended this approach, starting with a unit interval that can be recursively sub-divided into 10 equal parts to represent decimal numbers like 0.37 and 0.173. Their article presented evidence that the number line provides a basis for understanding that 0.37 is actually a larger number than 0.173, something children first encountering decimal numbers often fail to understand. By teaching children that each successive digit to the right of the decimal point corresponds to each successive partitioning of the number line, so that the first digit specifies where the number lies within the top-level partitioning, and subsequent digits specify where it falls within subsequent partitions within the partition specified by prior digits, it became possible for children to see that 0.37 lies further to the right of 0.173, so that 0.37 corresponds to the larger number. Moss and Case (1999) went on to show that their approach led to greater success in mastering basic aspects of decimals and fractions than standard approaches to teaching about these aspects of number.

### 1.2 The unit circle as an integrative conceptual structure for trigonometry

**Figure 1:**
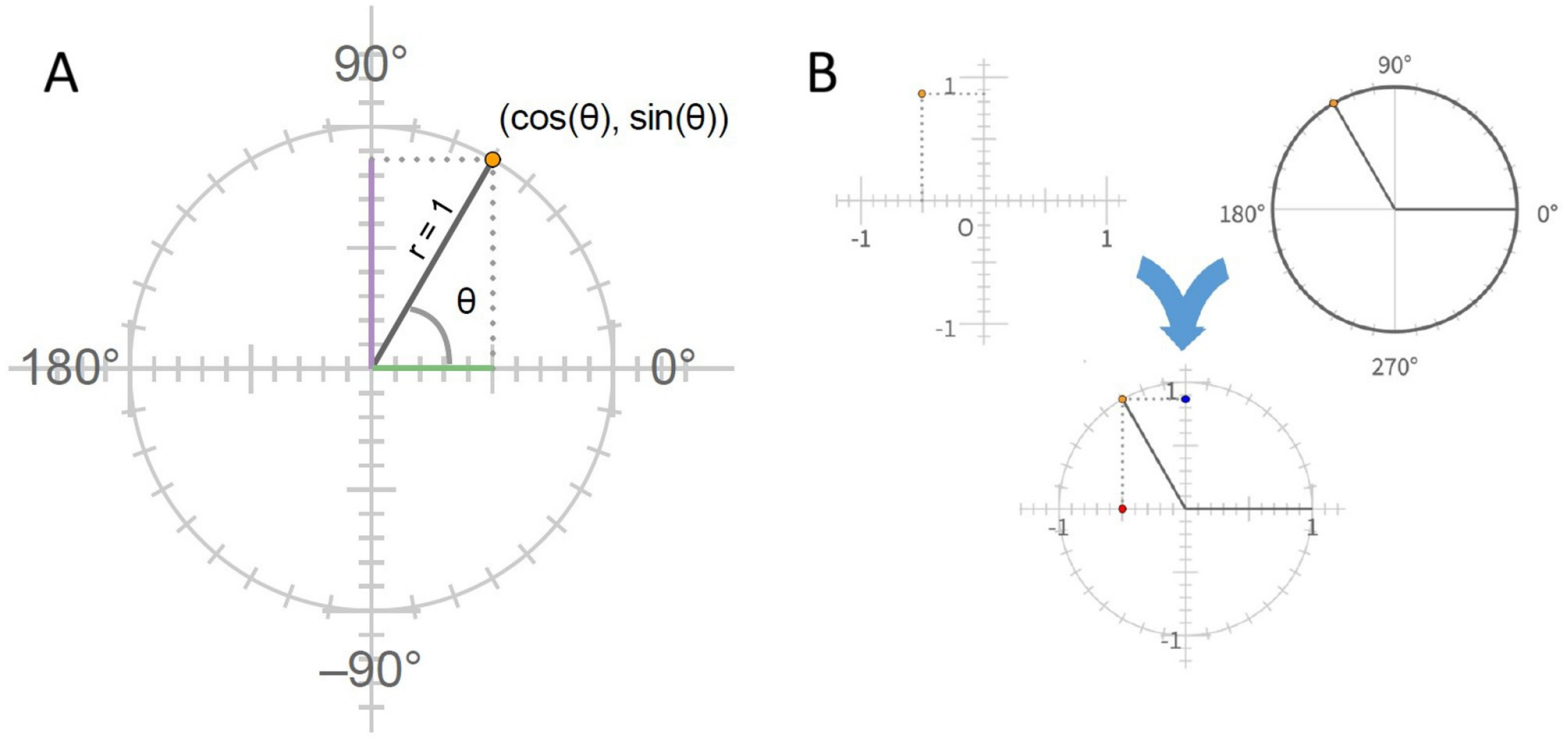
**(A)** The unit circle. In triangles, cosine is defined as the ratio of the length of the side adjacent to the angle (shown in green in the figure) to the length of the hypotenuse, and the radius of the unit circle is equal to 1. The ratio then reduces to the length of the horizontal side. Similarly the sine ratio reduces to the length of the vertical side (shown in lavender). On the unit circle, these quantities correspond to the values of the x and y coordinates of the point where the terminal side of the angle intersects the circle. **(B)** The unit circle is a conceptual structure in trigonometry that integrates the system for representing positions of points on the X-Y coordinate plane with a system for representing positions around the circumference of a circle.

In Mickey and McClelland (2025), we described a visuospatial structure called the unit circle that, we propose, can play a similar role to the mental number line for understanding trigonometric concepts and relationships. Here we briefly review how the unit circle (shown in Figure 1A) provides an integrated conceptual structure for representing the meanings of trigonometric expressions involving the sine and cosine of a numerical quantity. This quantity, typically represented by the symbol *θ*, is conceived of as corresponding to the position of a point on the unit circle, and the cosine and sine of *θ* are conceived of as representing the horizontal and vertical coordinates of this point on the Cartesian plane on which the unit circle is centered. The integrated conceptual system here combines the Cartesian system for representing the positions of points on the plane—itself combining two decimal number lines that extend in two directions from 0—with a system for representing the positions of points on the circumference of a circle with radius equal to 1 (see Figure 1B).

**Figure 2:**
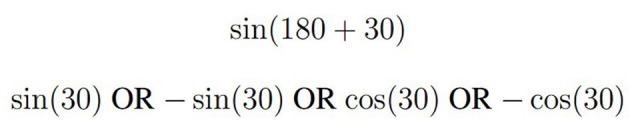
An example problem from the trigonometric identity test used in Mickey and McClelland (2025) and in the current study. The test consisted of 40 such problems, as described in *Methods* Section 2.3.

In Study 1 of Mickey and McClelland (2025), we found that many undergraduate students at Stanford University, all of whom had had exposure to precalculus trigonometry in prior coursework, performed poorly solving a set of 40 trigonometric identity problems like the one shown in Figure 2. However, those participants who reported relying on the unit circle were more likely to solve these problems correctly. As one striking example, students who did not report relying on the unit circle performed particularly poorly on a basic trigonometric relationship fundamental to precalculus trigonometry, captured by the expression cos(−*θ*) = cos(*θ*): On problems requiring them to chose an expression equivalent to the expression cos(−*θ*) (for some specific value of *θ*, such as 30 degrees), these students were more likely to chose −cos(*θ*) rather than the correct alternative, which is simply cos(*θ*). In contrast to these students, those who reported relying on the visuospatial conceptual model provided by the unit circle were much more likely to answer the cos(−*θ*) problem correctly, and performed better overall on the larger set of trigonometric identity relationships probed in our study.

**Figure 3:**
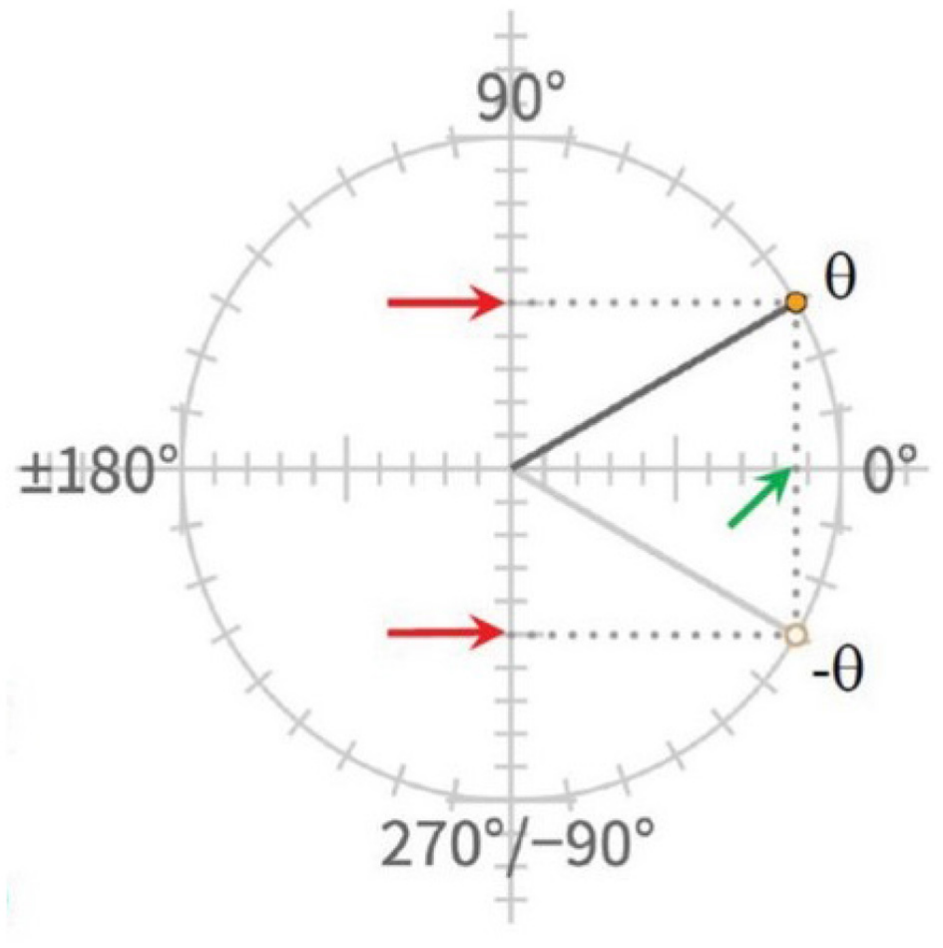
The unit circle makes it possible to see why cos(−*θ*) = cos(*θ*) (green arrow) while sin(−*θ*) = − sin(*θ*) (red arrows), when conceived as relationships between the *x* (horizontal) and *y* (vertical) coordinates of points on the circle reached by rotations in the positive and negative directions from the 0 point, where the unit circle intersects the positive *x* axis.

To aid the reader's understanding of the usefulness of the unit circle, we briefly describe how grounding trig identities in it can support correct understanding of apparently arbitrary and seemingly contradictory relationships. The identity cos(−*θ*) = cos(*θ*) already mentioned, together with the identity sin(−*θ*) = −sin(*θ*) illustrate this point (see Figure 3). As purely symbolic expressions without such grounding, it is difficult to see why one has a minus sign on the right hand side and the other does not. However, once one understands how the elements of these expressions relate to the coordinates of points on the unit circle, it is possible to see what each of these expressions means and why they differ.

On the unit circle, angles correspond to positions reached by rotations of points around the unit circle, starting from the point where the circle intersects the positive, right-hand side of the *x* axis. Positive angles represent rotations in the counter-clockwise direction, and negative angles represent rotations in the clockwise direction. The cosine of an angle corresponds to its horizontal displacement from the origin of the circle, visualized in the figure as the projection of the point on the *x* axis of the Cartesian coordinate system, while the sine of an angle corresponds to the point's vertical displacement from the origin, visualized as the point's projection onto the *y* axis. Given the conventions of the Cartesian coordinate system and of the representation of positions of points in degrees, it becomes apparent from looking at the diagram that the *x* coordinate of the position one reaches by rotating an amount in the negative direction (−*θ* in the figure) is the same as the *x* coordinate of the position one reaches by rotating the same amount in the positive direction (*θ* in the figure), which is what it means to say that cos(−*θ*) = cos(*θ*) within the integrated conceptual system provided by the unit circle. In contrast, the *y* coordinates of the two positions are not the same; while they are the same distance from the origin along the *y* axis, they are in opposite directions, corresponding to the relationship sin(−*θ*) = −sin(*θ*). Similar points apply to other trigonometric identities, such as the one shown in Figure 2, which tests for a student's understanding of the identity sin(180 + *θ*) = −sin(*θ*). This expression, when grounded in the unit circle, and coupled with the knowledge that a rotation of 180 degrees takes a point half way around the unit circle, captures the fact that the vertical position of any point halfway around the circle from another point is the same distance from the origin as the original point but in the opposite direction. In general, mathematical expressions that seem arbitrary without grounding in the unit circle become meaningful if understood as expressing relationships between the coordinates of positions reached by rotations of a specific extent and direction around the circle from specified starting positions.

Based on our finding in Study 1 of Mickey and McClelland (2025) that students who relied on the unit circle preformed better in solving trigonometric identity problems, we went on to explore the benefits of using this to ground instruction in trigonometric identities. In Study 2 of that article, conducted with students from the same Stanford undergraduate population used in Study 1, we found benefits from a brief lesson presenting these identities as expressing relationships between coordinates of points on the unit circle, over and above the benefits provided by a purely formal, rule-based lesson. While students in our population showed equal improvements on relationships explicitly taught in both types of lessons, only students receiving the grounded lesson showed an improvement on transfer problems not explicitly taught. This supported our view that instructional materials emphasizing the grounding of trigonometric relationships in the unit circle could indeed contribute to better learning outcomes.

### 1.3 Exposure to vs. reliance on the unit circle

The key challenge we found ourselves facing arises at this point. Exposure to the unit circle is very common in precalculus trigonometry, yet many students apparently do not learn to rely on it. Precalculus trigonometry textbooks by several different authors mention the unit circle in their titles, and the Stanford undergraduates in Study 1 of Mickey and McClelland (2025) were generally familiar with it; they gave a median rating of 4 out of 5 corresponding to “quite a bit” of exposure to the unit circle. However, self-reported *reliance* on the unit circle was correlated with accuracy on the trigonometric identity problems in Study 1 of Mickey and McClelland (2025), but self-reported *prior exposure* was not. Furthermore, in Study 2, we found that the participants who performed poorly in the pre-test prior to receiving our brief unit circle lesson showed little or no benefit from it; only those students who performed reliably above chance in the pretest showed a benefit. This suggests that mastery of the integrated conceptual system provided by the unit circle may be challenging for many students, leading them to rely on rote memorization of formulas or other less effective learning strategies.

The idea that mastery of the unit circle can be challenging for many students was further supported in a pilot study with a group of high-school seniors enrolled in a precalculus class at a public high school in the affluent community near our university. Most of these students were university bound, though not necessarily in STEM fields. We judged that they fell primarily in the second and third quartiles in mathematical ability of students in their school, and that most would be above average in mathematical ability in a national US sample of students in their grade level. We invited these students, as they were completing a six-week instructional unit on precalculus trigonometry, to complete the unit-circle-based condition of Study 2 of Part I. As did the students in our Stanford sample, these students first attempted to solve the set of 40 trig identity problems, each containing a randomly chosen specific angle, (as in the example in Figure 2), then completed the brief unit circle lesson used in that study, and then attempted to solve the set of 40 identity problems again with different randomly chosen specific angles. Strikingly, all but one of the students who participated performed near chance on problems other than the most trivial identity problems (sin(*θ* + 0) = sin(*θ*) and cos(*θ* + 0) = cos(*θ*)) in block 1, prior to the brief unit circle lesson, and as a group they showed only a 6% improvement over their block 1 performance after the lesson (Mickey, 2018). These results suggest that their high-school curriculum was less successful than we had hoped in creating a basic understanding of trigonometric relationships even though it included exposure to the unit circle, and that, even with several weeks worth of prior exposure to the subject, a brief lesson grounding trigonometric relationships in the unit circle is not sufficient to allow many students to master and exploit the conceptual model provided by the unit circle in their reasoning.

### 1.4 Why do so many students fail to master the unit circle?

Both in our Stanford sample used in the studies of Mickey and McClelland (2025) and in the students in our pilot study, failure to rely on the unit circle was common. Why? Here we consider two kinds of factors that, we suggest, may contribute to this outcome.

#### 1.4.1 Challenging aspects of the unit circle framework

The unit circle framework itself may contribute to the difficulty students face in mastering it: it involves many arbitrary and potentially confusing conventions that partially conflict with each other and in some cases conflict with prior experience.

First, consider the conventions of representing the positions of points on the Cartesian plane. These conventions involve using pairs of numbers, such as (3, −7) to represent such positions. These numbers must be understood as referencing positions relative to a special point, the *origin*, at 0.0 (see Figure 1B, upper left). The first corresponds to the distance of the point in the horizontal direction from the origin, which, by convention, is positive for positions to the right of the origin and negative for positions to the left. The second corresponds to the distance of the point in the vertical direction from the origin, which, again by convention, is positive for positions above the origin and negative for positions below. If a student is unsure of any of these conventions they may fail to follow statements that refer to the relationships among these points. Indeed, in materials we developed for assessing students understanding of the Cartesian plane, we found that students often made errors in placing points on the Cartesian plane, reflecting only partial mastery of these relationships. Second, consider the conventions for representing values of the variable *θ*. To begin with, when students first encounter this variable in precalculus trigonometry, they may think of it as measuring the extent of the angle between two line segments based on previous engagement with angles in introductory geometry, whereas for use of the unit circle it important to see it as referencing a position on the circle that results from a rotation in one direction or another from a reference point. Next, like the horizontal and vertical coordinates of the Cartesian plane, *θ* has an origin and a direction, both of which are arbitrary. The origin falls at the point on the Cartesian plane where the unit circle intersects with the positive (right) side of the horizontal axis through the origin of the Cartesian plane, and the positive direction is counter-clockwise. Both of these conventions not only seem arbitrary; they also run counter to experience with clocks, where the origin is above, not to the right, of the center of the clock, and where the direction of rotation corresponding to increasing numbers is clockwise (down to the right from the origin) rather than counter-clockwise. Furthermore, positions around the circle are represented by numbers in the range ±360deg, with several of these numbers – particularly 0, ±90, and ±180 being significant landmarks often referenced in trigonometric expressions, reminiscent of the landmarks at one half and one-quarter of the number line that play a role in students understanding of the mental number line (Moss and Case, 1999; Barth and Paladino, 2011). Finally, consider the functions cos and sin that relate these coordinates to each other. The first designates the horizontal coordinate of a point on the unit circle, while the second designates its vertical coordinate, another convention that can seem completely arbitrary. Uncertainty and/or lack of fluency with respect to any of these conventions should be expected to make it difficult for a student to rely on visualization, raising the barrier to reliance on the integrative conceptual framework provided by the unit circle.

The reader might think that unit-circle trigonometry is particularly demanding with respect to these conventions, and there may be some truth to this. We would argue, however, that such issues arise in other branches of mathematics as well. The conventions of the decimal number line discussed earlier in relation to the work of Moss and Case (1999) have arbitrary elements too, including alignment of the rightward direction with increasing numerical value and the relationship between digits in a numerical value like 0.173 and successively embedded partitions of the interval between 0 and 1 along this line. As another example, in statistics and in the application of statistical concepts to signal detection theory within psychology, the expression N(μ,σ) references the mean and standard deviation of a normal distribution visualized by the position and spread of a bell-shaped curve. Furthermore, the expression Φ (*x*) for x~N(μ,σ) references the cumulative area under this curve from −∞ up to *x*, or the area under the graph of this curve to the left on *x*. Again there are arbitrary and unfamiliar conventions in play in gaining a grounded understanding of the meanings of these expressions. Our point here is that the need to master seemingly arbitrary relationships between visuospatial and symbolic representations may be relevant widely throughout mathematics and its application in quantitative theories in scientific fields, and access to an understanding of the relevant concepts and theories may often depend on mastery of these relationships.

#### 1.4.2 Limitations of classroom practices and textbooks

Next we consider potential limitations of classroom instructional practices and textbooks. As cognitive scientists, we lack deep backgrounds in these practices, and we claim no extensive knowledge of relevant textbooks. However, the experience two of us (KM and JLM) had in the classroom and with the text book (Foerster, 1990) used by our high-school pilot study participants during the six-week period when trigonometry was covered in their precalculus class, starting about one month into their fall semester, suggested to us that their may be substantial room for improvement. It was apparent to us that many of these students had reached the class without sufficient background knowledge, effort-oriented mindset, or associated skills to be fully prepared to engage with the material being covered in the class. Also, while the teacher assigned readings and homework from the textbook and walked through the relevant concepts and homework problems in class, we noted limitations in both the presentation of content and management of student learning activities: While both the textbook and classroom presentation included symbolic and visuospatial elements including the unit circle, there was little systematic effort in either the book or the classroom to ensure the students engaged with the relevant visuospatial concepts or that they mastered the conventions involved in these relationships before proceeding. It seemed to us that both the textbook and the classroom presentation emphasized rote memorization of tables of abstract symbolic formulas rather than full engagement with the visuospatial models. The visuospatial models were often alluded to only briefly in the text, and many relationships were justified based on purely symbolic manipulation according to algebraic manipulations and appeals to rules listed in tables. It also seemed clear to us that many of these students did not read assigned sections of the textbook or complete the assigned homework prior to class and were not always attentive in the classroom. Finally, the classroom practices were not very demanding, and evaluation centered on quizzes and exams, making it easy for students to avoid steady engagement with the material, and then get an acceptable grade based on lenient grading of these assessments.

### 1.5 Toward the development of a curriculum for engendering reliance on the integrative conceptual framework provided by the unit circle

The considerations reviewed above led us to ask: would it be possible to create a curriculum that would successfully support robust mastery of the integrative conceptual framework provided by the unit circle in students with no prior familiarity with precalculus trigonometry? Through an iterative design and conceptualization process, undertaken in consultation with colleagues in educational psychology at Stanford and elsewhere, as well as members of the mathematics teaching staff at a West-Coast based Charter School network, we worked to develop a curriculum that we hoped would achieve this goal.

Our goal was to develop a curriculum that would address the challenges posed by the unit circle and the instructional pitfalls described above, to support robust learning in university-bound high school and community college students without prior knowledge of the sine and cosine functions beyond their definitions in right triangles. As a target, we aimed to create a curriculum that would allow 75% of students with sufficient prior knowledge to show evidence of robust learning, using the same 40-item trig identities test we used in Mickey and McClelland (2025) to assess learning outcomes. As a measure of robustness, we focused on retention after a delay rather than transfer to held-out items, taking the view that achieving robust mathematical ability rather than fragile knowledge that fails to survive beyond an immediate post-test would benefit from generalized practice that was not restricted to a subset of examples. We therefore evaluated performance both 48 hours after completing our lessons, and (in a subset of participants) at least two weeks after the first post text. Also, while we considered including a comparison lesson condition, we decided after consideration to focus on achieving a single lesson that was successful relative to a no-lesson control, since it seemed to us, as discussed further below, that a wide range of factors jointly contribute to success in helping students achieve a robust, grounded basis for good performance in a new set of mathematical reasoning skills, and that it would be difficult to devise a good comparison condition that manipulated some of these without introducing confounds with others.

The choices described above all reflect the need to strike a balance between pragmatic and aspirational goals, and we acknowledge that our materials reflect our own subjective choices and are confined to a single example subtopic within the broader subject of trigonometry and the broader field of mathematics. Yet we felt that the effort to achieve it, if successful, would be worthwhile, because the kinds of factors limiting the success of the students learning precalculus trigonometry in our high school sample seemed broadly relevant to many aspects and levels of the mathematics curriculum as it is encountered by students all over the world.

#### 1.5.1 Psychological and didactic principles of the trig academy curriculum

As we developed our curriculum, we were guided by a set of principles that have been established within in the cognitive and educational science literatures. We now present these principles, beginning with the core starting principle on which we based our curriculum:

- **Grounding in an integrative conceptual framework**. Grounding mathematical concepts expressed in words and equations to their underlying meaning in an integrative conceptual framework serves as an effective starting place for building a robust understanding of mathematical relationships.

Prior work of Case et al. (1996) and others along with our own experiments in Part I (Mickey and McClelland, 2025) underscore the importance of grounding in a integrative conceptual framework, with our work in Part I establishing that the unit circle provides a useful conceptual framework for understanding the trigonometric identity relationships involving the sine and cosine functions we focus on throughout this project. Accordingly, we aimed to design a curriculum based on relating these trigonometric expressions to their meanings, conceived of as expressing relationships among the horizontal and vertical positions of points on the unit circle.

A second principle that we relied on in designing our curriculum grew out of a key finding of Study 3 from Part I of our investigations.

- **Grounding applies to both rules and procedures**. Grounding gives meaning to statements of rules, increasing their memorability and generalizability, and also allows the construction of procedures that support reasoning about mathematical relationships.

In study 3 of Part I, we observed that our grounded lesson led to increased self-reported reliance on both rules and the unit circle. Further analyses of think-aloud protocols with students in this study suggested that this pattern could be understood in terms of the idea that participants were often relying on **grounded rules**. By a grounded rule, we mean a generalizable statement about a trigonometric relationship that is linked to the grounding of these relationships in the integrated conceptual structure. An example from the protocol of one of the students in this study provides an example of such a grounded rule: When describing how they solved a trig identity problem with the probe expression cos(20 + 180), this student said “I know it's going to go basically shift right across that [rotates hand]... it's just pretty much negative of whatever the other angle is”. We take this statement as expressing the fact that the relationship generally holds, referencing (albeit elliptically) the fact that a 180 degree rotation takes a point on the circle to the point opposite the starting point on the other side of the circle, connecting this with the use of negation to specify that the horizontal position reached by such a rotation is the same distance in the negative direction from the horizontal position of the starting point before the rotation. The insight that successful students often rely on grounded rules led us, in the development of our materials, to both (a) link trigonometric expressions to rotations and positions and on the circle and the corresponding horizontal and vertical coordinates and (b) to describe, and encourage learners to describe, these relationships in general terms.

Based on the challenging aspects of the unit circle and the limitations in classroom instruction and textbooks discussed above, we realized that creating a set of materials that would allow most students to succeed would require us to build our materials in accordance with a larger set of psychological and pedagogical principles, which we first list and then discuss in turn.

- **Grounding symbols in perception and action**. Direct engagement with and manipulation of a concrete visuospatial instantiation of the conceptual framework and bi-directional mapping between this concrete instantiation and linguistic and symbolic representations can support mathematical understanding and reasoning.
- **Prior mastery of components**. All students should have sufficient prior mastery of component concepts and relations before exposure to new concepts and relations that build upon them.
- **Active engagement**. Learning materials should ensure student engagement by requiring them to use the material presented to them to perform specified tasks that depend upon it.
- **Immediate feedback**. Student learning should be supported through actively monitoring responses and providing immediate feedback on response accuracy at each step of the way, so that students can immediately identify gaps and misunderstandings and re-engage with the material to address these gaps as they arise.
- **Fluency through practice**. Fluency with the conventions of a conceptual system is necessary to allow the grounding of mathematical ideas strongly enough to allow them to support reasoning and problem solving. It should be expected that students will need practice to establish sufficient fluency.
- **Progressive formalization**. Students should be introduced to concepts using familiar terms they already understand before introducing novel and seemingly arbitrary formal terminology.

The principle of **grounding symbols in perception and action** arises from the work of many others, including the writings of Lakoff and Nunez (2000) and the findings of Nathan et al. (2021) and other studies directly demonstrating that making gestures congruent with geometric ideas facilitates geometric reasoning. We relied on this principle as a central design constraint for our curriculum. We see this approach as largely consistent with the principles of the cognitive theory of multimedia learning (Mayer, 2024) as well. Specifically, we designed our curriculum so that it pairs language-based with visuospatial materials at every step of the way, requiring the learner to read the coordinates of points and report out relationships between points on the Cartesian plane and the unit circle; to place points at specified locations on the plane and the circle and consider them in relation to other points; and to manipulate points on the unit circle by grabbing and moving them with their mouse, and then to observe and report the consequences of these manipulations. In accordance with the importance of **grounded rules as well as procedures**, we stressed the generality of the relationships students observed, providing them with statements about these relationships and asking them to evaluate them.

The importance of **prior mastery of components** is nearly universally agreed upon, but ensuring that it is adhered to in practice may not be; as mentioned above, our observations in the classroom suggested to us that many students end up being exposed to new concepts without ensuring that they have mastered their components. This principle factored into our design in two ways: First, we sought to identify mathematical abilities that, if absent, prevented success. Second, we sought to include materials that would ensure facility with the component concepts that our unit-circle materials build upon (namely, the Cartesian plane and the circular coordinate system).

The principle of **active engagement** can be linked to the ICAP framework of Chi (Chi and Wylie, 2014), and is consistent with the principles of many computer-based tutoring systems including Anderson's cognitive tutors for mathematics and computer programming (Anderson et al., 1995) as well as Mayer's theory of multimedia learning (Mayer, 2024). The importance of **immediate feedback** is well-established in the conditioning and learning literature, and details of reward timing may play a role in ensuring that relevant signals are available to stamp in plastic changes in synaptic connections that underlie gradual, implicit learning underlying fluency (Maddox et al., 2003). In accordance with these principles, our materials required students to answer questions or take actions, and provided students with accuracy feedback and the requirement to correct errors before proceeding.

The principle of **fluency through practice** draws heavily on findings in cognitive psychology establishing that practice results in greater speed, accuracy, automaticity, and robustness to the effects of degradation due to competing attention demands (Schneider and Shiffrin, 1977) or even effects of neuro-degenerative disease (Ralph et al., 2001). When new abilities are initially acquired their use is slow and attention demanding, but they become less and less so with practice, to the extent that they can become sufficiently automatic (as in the case of word reading) to interfere with other tasks (such as naming the color of the ink in which a word is written, if the word and the ink color are not congruent MacLeod and Dunbar, 1988; Cohen et al., 1990). To address this, our curriculum included practice and required demonstration of a pre-requisite degree of facility with the Cartesian and polar coordinate systems prior to relating them to each other, as the sine and cosine functions do.

The principle of **progressive formalization** arises from research focused on developing best practices for enhancing student learning in STEM (Romberg, 2001). Nathan (2012) emphasizes the importance of introducing formal notation gradually, after the relationships the formalism introduces have already been considered. This approach contrasts with a formalism first approach, which Nathan (2012) argues often appears in mathematics, science, and engineering education, and he describes a body of empirical evidence supporting the idea that formalisms should be introduced after students have come to appreciate the relationships that formal concepts express. This idea can be linked with the idea from human memory research that building semantic associations between items to be learned supports more durable and robust learning (Craik and Lockhart, 1972; Bower, 1970) and resonates well with the principle of fluency through practice.

Taken together, fluency through practice and progressive formalization played a central role in our curriculum, leading us to introduce the terms sine and cosine and formal expressions like sin(30) and cos(30) only after familiarizing students with the idea that positions on the unit circle specified in degrees correspond to the horizontal and vertical components of the positions of these points relative to the center of the circle. Similarly, our curriculum emphasized concrete instances of specific positions before stating more general relationships, and referred back to the relevant more basic concepts and their grounding in linking positions on the circle to coordinates of points on the plane even when using formal expressions. Only after students demonstrated a degree of proficiency in this did we then encourage them to bring circle positions and their coordinates to mind for themselves and to continue to engage with the grounded visuospatial model as they attempted to answer trigonometric identity problems presented only in the form of symbolic expressions.

Beyond all of the principles mentioned above, we also recognized that engagement, motivation, attitude, mind-set and framing are all crucial in achieving successful student outcomes. These factors are becoming more prominent in evolving cognitive theories of learning (Mayer, 2024), and they came up repeatedly in our interactions with classroom teachers and mathematics education team leaders in the charter school network that we engaged with as we developed our curriculum. To address these issues, we created a set of framing materials, including an introductory overview stressing the idea that mathematical ideas can be understood as meaningful relationships in a conceptual system and providing an overview of the ideas that would be covered as well as short introductions to each of the six units of our curriculum. These materials included animations using colorful chess pieces as avatars and messages encouraging students to take their time to ensure understanding before moving on and to connect new concepts to more familiar conceptual components.

It is important to note some elements that our curriculum did not include. We did not link the Cartesian plane or the unit circle to actual instances of real physical situations, and we only briefly noted the many practical uses of trigonometry in real-world situations in the introduction. Furthermore, we considered only the sine and cosine functions, and even with these, our materials include only a subset of the relationships among them. We consider these obvious limitations that could be addressed within the same set of principles we have described (see Discussion for further consideration). Finally, our curriculum is completely focused on individual student learning though interactive engagement with our curriculum on line, and, in our controlled comparison study with community college students, was administered without any person to person contact between ourselves and the participants in the study. The study, therefore, misses out on exploiting the benefits of student-teacher or student-student engagement and support, which likely contribute substantially to positive outcomes in mathematics learning.

In sum, we sought to create a curriculum that would go as far as reasonably possible in encouraging individual learners to adopt a conceptual rather than memorization-based approach to understanding trigonometric relationships on the unit circle, encouraging fluency with the component conceptual systems represented by the Cartesian plane and the circular coordinate system before engaging with the integration of these in terms of the sine and cosine functions, presented as expressions linking positions on the unit circle to horizontal and vertical components of positions on the plane, before progressing to a consideration of the relationships between such positions captured by trigonometric identities.

#### 1.5.2 Curriculum development, aptitude and math skills test battery, and preliminary studies

We developed a six-lesson unit-circle-based curriculum called *Trig Academy* according to the considerations mentioned above over a 2-year period. The curriculum was initially intended for high-school students, and was initially developed with participants recruited from a charter school network in the San Francisco bay area. However, our high school population was limited in size, and we gained access to a much larger population of community college students participating in a research experience program as part of a Psychology course requirement. Using students from both populations, we refined our materials, ending up with a six-lesson curriculum that seemed to work equally well for high school students from the charter school network and the community college students who elected to participate.

In parallel with refining the materials, we developed an aptitude and math skills (AMSA) test battery to identify factors predictive of success with our curriculum. This battery, described in Supplementary Section 1.1, was administered together with several additional questions assessing math attitudes and prior mathematics coursework to over 1900 Community College students who completed these materials as part of a course requirement, allowing us to assess the correlations among the elements of the battery and identify their factor structure as described in Supplementary Section 1.2.

In our preliminary studies with students from both populations, we found that performance on the curriculum was partially explained by performance on a subset of the AMSA tasks (See Supplementary Section 1.3), and we used performance on a subset of the components of the AMSA to set a criterion for inclusion in the current study to limit participation to students who were more likely to succeed, as described below. Data and a fuller description of the findings from our preliminary studies is available on line as are the full results of the study reported below (see *Data Availability Statement*).

## 2 Methods and materials

The current study sought to confirm the efficacy of our curriculum in a controlled comparison study, using a sample from the community college population. We employed a two group design that allowed us to assess the effectiveness of the lessons in promoting acquisition of proficiency solving trigonometric identity problems and to assess the robustness of the proficiency acquired as measured by retention after a 2-3 week delay.

### 2.1 Participant population, recruitment, and screening criteria

Participants were recruited from a larger pool of students taking introductory psychology classes at three community colleges in Santa Clara County, California. Students from this pool were invited to complete our AMSA test battery for credit toward completion of a research participation requirement. Students who took the AMSA and who met the inclusion criteria stated below were given the opportunity to sign up to participate in the study for payment in the form of a $75 Amazon give card. The study was described as requiring about 8 hours of their time distributed over a 6 week period, and students were told that the purpose of the study was to help find better ways of teaching math concepts.

Inclusion in the study was based on performance on four of the tests in the AMSA called supported math (SM) tests: placing points on a Cartesian plane given X-Y coordinate pairs, marking angles given a number between 0 and 360 in degrees, answering basic questions about trigonometric ratios on a right triangle, and reading approximate values of the sine and cosine of points on the unit circle, with each task being performed with an on-screen reminder of the relevant conventions as support for the student. We set a screening threshold of 50.0% correct averaged across the four tests. To avoid participants with prior knowledge of trig identities, we excluded participants scoring at or above 67% correct on a short (12-item) set of trig identity problems included in the AMSA^1^.

### 2.2 Study design

We divided our participants into two groups: a lesson early (LE) and a lesson late (LL) group. Each participant participated in two within-study assessments in which they obtained a trig identities score by completing the forty-item trigonometric identities (TI) test used previously in Mickey and McClelland (2025). We call these tests TI1 and TI2. Participants in Group LE competed a set of lesson materials over a 2-week period, then waited at least 48 hrs before taking TI1, while participants in Group LL simply waited for a similar period before taking TI1 (see Table 1). Thus a comparison of performance of the two groups on TI1 allows a between-participants assessment of the effects of completing the lesson materials on performance on our trig identities test. After TI1, participants in group LE waited for at least 2 weeks, while participants in group LL completed the lesson materials and waited at least 48 hrs before completing TI2. This second phase allows assessment of retention in the LE group and of within-participant improvement from TI1 to TI2 in the LL group.

**Table 1:** Timeline of tasks for the Lesson Early (LE) group and for the Lesson Late (LL) group, where TI1 and TI2 represent the Trigonometric Identities test at timepoint 1 and 2, respectively.

**Group**	**Time within experiment**			
	**Week 1-2**	**Week 3**	**Week 4-5**	**Week 6**
LE group:	Lesson materials	TI1	(Delay)	TI2
LL group:	(Delay)	TI1, framed as pre-survey	Lesson materials	TI2

Within each test, half of the problems were completed in a first block of trials, with the support of a unit circle tool available in the testing window, allowing students to place up to two points on the unit circle to visualize their projections onto the horizontal and vertical axes through the circle. The other half of the trials were completed in a second block with a blank screen instead of the circle tool.

In the analyses described below, we only consider students who performed above our screening threshold on the pre-assessment and who completed the entire study. Twenty participants in the LE group and 19 participants in the LL group met these criteria and were included in the analyses.

### 2.3 Trig identities test

The trig identities test consisted of 40 items like the example shown in Figure 2. Each problem included (a) a probe expression shown in the first row of the problem display and (b) a set of four alternatives separated by the word OR, one of which is equivalent in numeric value to the probe expression. The probe expressions were constructed by selecting one of the trigonometric functions sin or cos, applied to a compound argument constructed by choosing an offset from the set {−180, −90, 0, 90, 180}, a specific angle chosen from the set {10, 20, 30, 40, 50, 60, 70, 80} and a sign (positive or negative) for the specific angle. The set of minimal expressions all contained the same specific angle used in the probe expression. Participants were instructed that the numbers represented angles in degrees, and that they should choose the alternative that was equivalent in numeric value to the probe expression. The test items covered all 40 possible combinations of the two functions, five offsets, two signs for the specific angle, and two orders of the angles (offset first or specific first) within the expression, with the specific angle chosen randomly for each problem for each participant and instance of the test.

### 2.4 Curriculum details

Our unit circle curriculum consists of six lessons, each containing many pages with explanatory text, questions requiring responses, and a response interface for student answers, including a manipulable unit circle tool as well as a menu for multiple choice items and a text box for numerical answers. The first lesson introduces the horizontal and vertical number lines and the Cartesian coordinate system. The second introduces the circular coordinate system using degree measure to specify positions. Subsequent lessons progress through relating the coordinate systems to each other by linking circular positions to their horizontal and vertical coordinates, then determining sine and cosine values of positions, then using compound expressions involving important landmark quantities (±90 or ±180 degrees) to find positions on the circle and determine sine and cosine values. The last two lessons require the student to engage with the task of comparing trigonometric expressions using unit circle visualizations. Earlier lessons foreshadow important concepts in simpler settings, and later lessons build on the content of earlier ones, with the goal of creating a comprehensive mental model students can use to ultimately visualize trigonometric expressions. The full set of lesson materials is available online at our Unit Circle Trigonomety repository^2^, and a publicly accessible web site^3^ allows anyone with a gmail account to access the entire set of materials interactively as they were experienced by the participants in our study.

All lessons were developed with an emphasis on creating a conceptual understanding of the meaning of trigonometric expressions linked to the visuospatial unit circle model in coordination with the standard Cartesian coordinate plane. The modules required students to interact with diagrams on the platform, and to use what they had learned to reason about trigonometric relationships. The materials emphasized meaning-making, encouraging students to reflect on what they were learning and what it meant, and to understand what they have learned about particular cases as examples of relationships that hold more generally, supporting the acquisition of grounded rules. We adopted the principle of progressive formalization, in which concepts are initially expressed in concrete descriptive terms, building toward gradual replacement of such terms with conventionalized symbolic terminology. For example, the vertical position of a point on the unit circle gradually transitions to the y-coordinate of the same point and eventually to the sine of the numerical expression corresponding to the point on the circle.

The platform required students to engage by posing multiple choice or fill in the blank questions as well as questions requiring students to mark positions in space either on the X-Y plane or on the unit circle. Students received immediate feedback for incorrect responses, which they were then required to correct. Each lesson was divided into sections on which students had to achieve 75% correct first responses to the questions in the section before they could proceed to the next section. The time students required to complete the six lessons was about 4–6 h.

To provide scaffolding and motivation, we created an introductory overview of our curriculum and coordinated introductions for each of the six lessons. The overview sought to introduce trigonometry, not as a confusing jumble of symbols, but as a system of concepts that capture meaningful relationships, which, if understood, would allow the student to evaluate the validity of expressions conceptually rather than to rely on rote memorization. The materials were meant to be visually engaging and to situate the learner (represented by a pawn) as moving through a cumulative progression corresponding to the sequence of units, culminating in receipt of a certificate of excellence, mastery or completion depending on the level of final performance achieved. Each unit's introduction revisited the overview, situated the learner in the progression to signify progress, and provided a brief preview of the topic to be covered and its role within the overall structure of the curriculum. These materials also stressed the importance of understanding each unit before progressing to the next, providing an explicit rationale for the requirement that the student achieve a target level of accuracy on probe questions within each unit before proceeding to the next unit, and encouraged the student to monitor their understanding and return to completed units for review when they felt uncertain.

## 3 Results

As we document below, we found clear evidence that our curriculum led many students to achieve proficiency solving trig identity problems, both in a between-group analysis comparing the performance of LE and LL groups on TI1, when the LE group had completed the six lessons while the LL group waited, and in a within-group analysis comparing the performance of the LL group on TI1 and TI2, taken before and after they completed the lesson. In addition, we found robust retention after a 2–3 week delay in a within-group analysis comparing the performance of the LE group on TI1, taken three days after completing our curriculum and TI2, completed at least 2 weeks after that. The statistical tests described in this section were pre-registered (Mickey et al., 2020) prior to any examination of the data. As discussed in Supplementary Section 2, we conducted each of these analyses twice, once without consideration of problem type, and once with problem type as a random factor. A consistent effect regardless of the inclusion of problem type as a random factor would indicate robustness of the effect. For each analysis, we assessed statistical reliability with both the Wald test and a bootstrap test, giving *p* values for each of 4 significance tests for each comparison of interest, shown in a separate table in Supplementary Section 2 for each of the three analyses described below.

### 3.1 Between group effect of lesson

Our first analysis asked whether the lesson-early group performed better in the first within study trig identities test than the lesson-late group. To assess this, we used a logistic mixed model to predict TI1 score for all participants, using lesson timing (LE group vs LL group), SM score, and their interaction. We included a random intercept for each subject. If the lesson enhanced student performance, we would expect a positive effect of lesson timing, where the LE group tends to achieve higher accuracy than the LL group. We are also interested in estimating the size of the main effect of SM score and the interaction of SM score with lesson timing.

**Figure 4:**
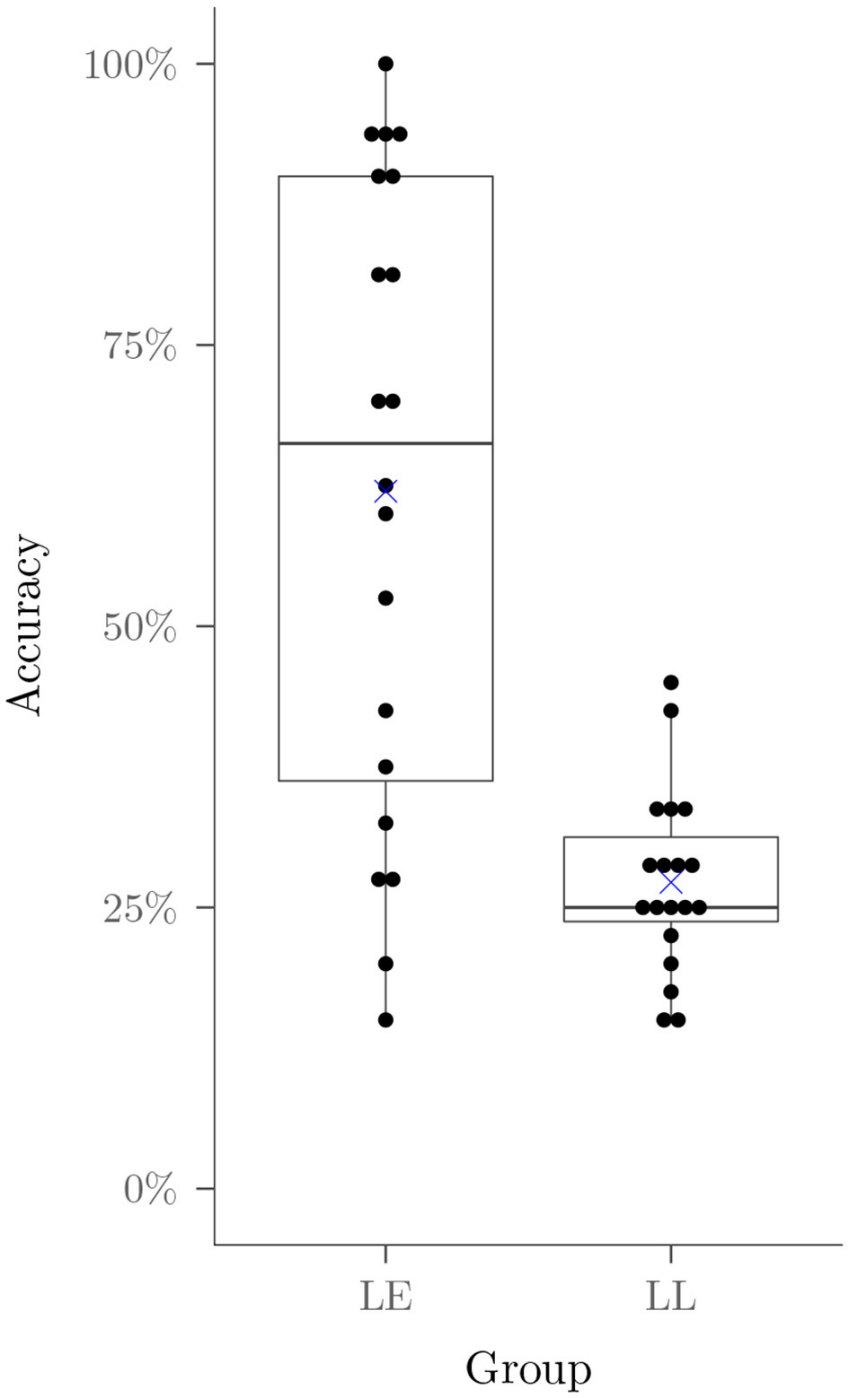
Accuracy on the first trig identities test for participants in the controlled comparison study. Participants in the Lesson Early group (LE) received the lesson prior to taking the test, while participants in the Lesson Late (LL) waited for a comparable period.

These analyses demonstrated robust statistical support for an effect of group (*p* < 0.001 in all four tests), as shown in Figure 4. Inspection of the distribution of scores for the lesson early group shows a wide range, with 13 of the participants in the LE group receiving post-test scores above the range of scores in the LL group. Neither the effect of SM score nor its interaction with group were statistically reliable (smallest *p*>0.385). The interaction, however, is based on a between group comparison, and may not be as sensitive to capturing a possible relationship between SM score and improvement due to the lesson as the comparison within the LL group analysis, to which we now turn.

### 3.2 Effect of lesson within the lesson late group

Next we considered whether the lesson-late group showed improvement from the first within-study trig identities test to the second within-study trig identities test, and the relationship, if any, of this improvement to each participant's SM score. To assess this, we considered the LL group only, using logistic mixed models with a random intercept and random effect of test timing for each subject with and without problem type as a random factor to predict TI score, using test timing (TI2 vs TI1), SM score, and their interaction, obtaining both Wald and bootstrapped estimates of *p*-value.

**Figure 5:**
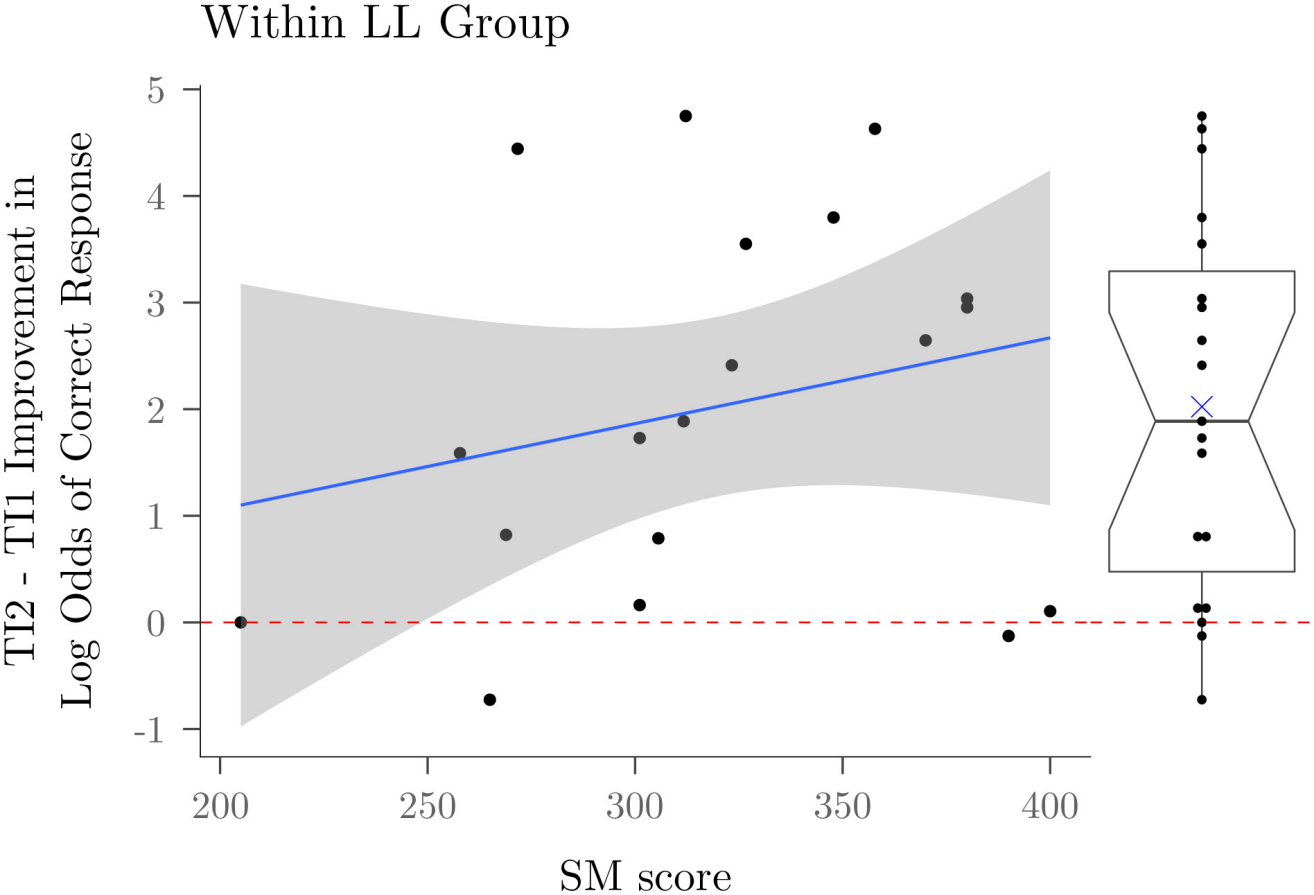
Improvement score between the first and second trig identities test for participants in the lesson late group. The left shows a scatterplot relating the magnitude of improvement to participants' score in the Supported Math component of the Aptitude and Math Skills Assessment battery. The right panel indicates the marginal distribution of improvement scores. The upper and lower bound of the notch represent the 95% confidence interval.

If the lesson enhanced student performance, we would expect a positive effect of test timing, where students achieve higher accuracy on TI2 than on TI1; if the effect varies with SM score, the interaction of test timing and SM score should be statistically reliable. The effect of time was highly reliable (*p* < 0.001 in all four measures). Inspection of the pattern of results, shown on the right in Figure 5, indicates that 12 of 19 participants showed substantial gains from the lesson, while 7 showed little sign of improvement. While the interaction of SM score with time was reliable (*p* < 0.001 in all four measures), the scatterplot on the left in Figure 5 shows that the association is relatively weak, and that some participants with lower SM scores showed clear improvement, while the two with the highest scores showed none. Lack of improvement for these individuals was not due to a ceiling effect—both scored in the near-chance range (below 50% correct) on TI1 along with all of the other students in the LL group (see Figure 4).

### 3.3 Retention within the lesson early group

Next we considered retention of learning from our lessons, and a possible relationship between SM score and retention, by examining whether scores of the LE group decreased between the first and second within-study assessment, using a similar analytic approach as described above. In fact we found no evidence of a performance decrease between TI1 and TI2 (*b* = 0.02 without problem type and *b* = 0.01 with problem type, smallest *p* = 0.717 in the four measures), and there was no evidence of a main effect of SM score. There was a weak trend toward an interaction (*b* = −0.9 and *b* = −0.12 with and without problem type as a random effect, *p* values ranging from 0.137 to 0.068), indicating that, if anything, those with lower SM scores showed better retention.

### 3.4 Relationship between circle tool use and accuracy

To learn more about the role of the unit circle in our lessons and whether students' engagement with it co-varied with their success, we examined the relationship between their use of the unit circle tool during the trig identities test and their accuracy in solving the test problems when this tool was available (in block 1 of the test) and when it was not (in block 2). As we now describe, this analysis uncovered strikingly different patterns across individuals in their frequency of tool use, together with a complex pattern in the relationship between tool use and accuracy.

We used a logistic mixed model, with subject as a random factor, to predict whether a student answered each trig identity test trial correctly, combining the results from all 39 participants on their first post lesson test (TI1 for the LE group, TI2 for the LL group). As a measure of circle use, we determined the proportion of trials in which participants clicked once or more on the external circle when it was available during each trial during block 1 of this test. We can distinguish the between-subject effect and the within-subject effect by using two different predictors in our model (Curran and Bauer, 2010): the student's mean circle use, and the student's circle use on a particular trial minus their mean circle use. We considered trials with and without the tool available (Blocks 1 and 2, respectively), and so included an indicator to reflect this difference. We then also included the interaction of block (corresponding to tool availability) and the between-subject effect of tool use. Finally, the model included random intercepts by subject and random slopes for the block effect and the within-subject effect of tool use to account for individual differences in these effects.

**Figure 6:**
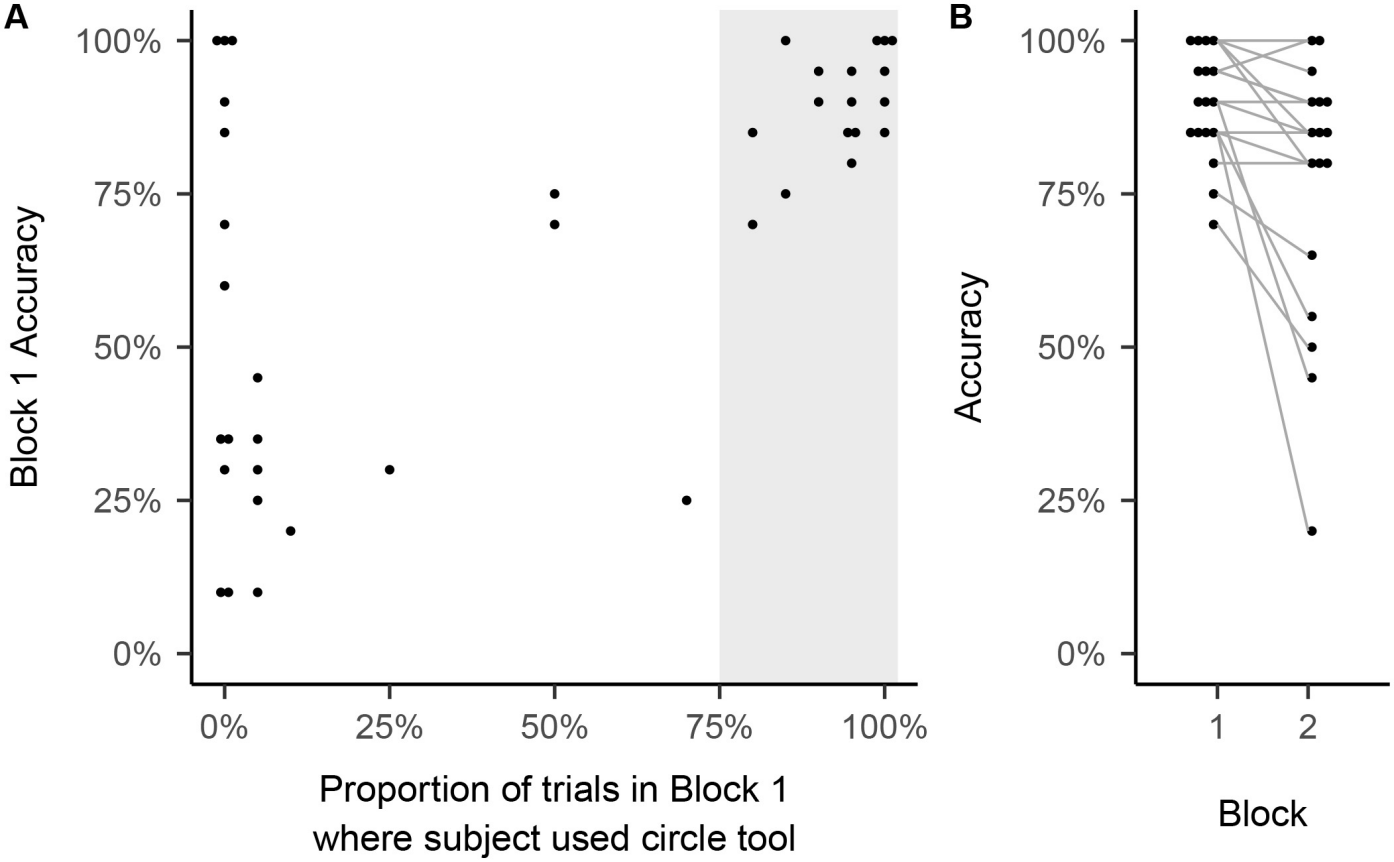
**(A)** Shows the relationship between use of the unit circle tool and accuracy in block 1 of each participant's first post-lesson trig identities test. **(B)** Considers those who interacted with the tool on at least 75% of block 1 trials [shaded region of **(A)**], showing the distribution of accuracy scores on block 1 and on block 2, with line segments joining each participant's block 1 and block 2 scores.

When the circle tool was available in block 1, tool use was highly predictive of accuracy characterized as a between-subject effect, *b* = 2.61, 95% CI [1.28, 3.94], *z* = 3.85, *p* < 0.001. Students who interacted with the external circle on more trials tended to have higher overall accuracy: Figure 6A shows that all those who used the circle tool on more than 75% of Block 1 trials responded correctly on at least 70% of the trials in this block. Conversely, most of those who used the circle on less than 25% or trials scored below 50% correct. Notably, however, 3 of these participants performed perfectly and 4 others exceeded 50% correct. Thus, while most successful learners used the unit circle tool on most trials, some who succeeded did not use the tool at all^4^.

When the circle tool was not available, the between-subject effect was marginally significant, though still positive, *b* = 1.42, 95% CI [−0.15, 3.00], *z* = 1.77, *p* = 0.077. However, the interaction of the between-subject effect and tool availability was significant, *b* = 0.59, 95% CI [0.06, 1.13], *z* = 2.17, *p* = 0.030. The interaction reflects the fact, displayed in Figure 6B, that many of those who relied on the circle in Block 1 showed a performance decrement in block 2, and for some the drop was considerable. Thus, for a subset of participants, mapping trigonometric expressions literally onto the unit circle may have been an important contributor to their success.

### 3.5 Predictors of successful outcomes from completion of the trig academy curriculum

Our goal for the Trig Academy was to create a curriculum that would lead to successful learning outcomes for 75% of students with sufficient prior math skills, as measured by the Supported Math subset of our Aptitude and Math Skills Assessment battery. Taking the 13 LE participants who achieved higher performance than any LL TI1 score from the between group comparison shown in Figure 4, together with the 12 LL participants who achieved significant improvement from the within group comparison shown in Figure 5, 25 of 39 participants demonstrated a benefit from the lesson, corresponding to a 64% success rate, and among those who did benefit, there was a considerable range of variation in the extent of benefit. Here we consider factors that may be associated with this variation.

Using data combined across a group of 62 high-school and community college participants who completed our Trig Academy materials before we conducted our controlled comparison study, we found that performance of the set of supported math tasks included in our aptitude and math skills assessment battery proved to be a statistically reliable predictor of a higher post-lesson score, *R*^2^ = 0.20, F(1, 60) = 14.84,*P* < 0.001. In further analyses based on the data from of this group (see Supplementary Section 1.3), we observed that the materials in the first four lessons in our curriculum were relatively easy for most participants, but the materials in the last two lessons, which required participants to compare sines and cosines of angular positions with different polarities and offsets from each other, proved to be considerably more difficult, and many students made more incorrect responses in this part of the curriculum. Furthermore, overall performance as measured by accuracy of participants' first response to the questions within these last two lessons was strongly correlated (*r* =.66, *p* < .001) with performance on our Trig Identities post-test, which also requires students to compare sines and cosines of angular positions with different polarities and offsets. This finding suggests that correctly determining and holding two such comparisons in mind may increase the cognitive demands of the lessons over and above the demands imposed by the material in the first four lessons, reducing the likelihood of success for many students.

Because participation in our controlled comparison study was restricted to participants whose SM scores were greater than or equal to 50% correct, the range of this variable was more restricted in the controlled comparison study we have reported in detail here. Within this more restricted range, we only found modest evidence that the SM score was associated with greater improvement in performance on our trig identities test, as discussed in Section 3.2. However, we did find one within-experiment measure that proved to be a useful predictor of success: As shown in Figure 6, active engagement with the unit circle when it was available in the first post-lesson block of our Trig Identities test was positively associated with performance, even when the tool was unavailable in the second block, suggesting that a proclivity to engage with it was associated with successful learning. This pattern of performance would be consistent with a virtuous cycle between understanding of and engagement with the visuospatial model; poorer performers may not have engaged with it because they failed, in spite of our didactic efforts, to establish a sufficiently strong link between the visuospatial model and the symbolic expressions our identities test required them to assess, so that it was of relatively little use for them to try to use it. The drop in performance from block 1 to block 2, where the circle was no longer available, suggests that active engagement with it supported correct performance for many of the students who did interact with it in block 1.

## 4 Discussion

Taken together, our experimental studies with Stanford University undergraduates described in Part I (Mickey and McClelland, 2025) of our two-part investigation and our intervention studies described in the present article provide evidence consistent with our hypothesis that grounding an understanding of trigonometric relationships in the unit circle can support robust learning. Our experimental studies in Part I found that students who reported relying on the unit circle performed better on trigonometric identity problems and that using the unit circle to ground a brief lesson in trigonometric identities produced a clear advantage in supporting transfer to untaught problems relative to a lesson presenting the same material using ungrounded formal rules. Building on this finding, and on an appreciation that relying on this grounding was a challenge for many students, we created Trig Academy, a curriculum aimed at helping students understand the meaning of trigonometric expressions as referencing the horizontal and vertical coordinates of points on the unit circle, using this as a foundation for understanding trigonometric identities as expressing relationships among such points. Rather than encouraging participants to simply memorize apparently arbitrary relationships such as cos(−*θ*) = cos(*θ*) but sin(−*θ*) = −sin(*θ*), we taught them to treat these expressions as capturing relationships between the horizontal and vertical coordinates of positions on the unit circle.

Our curriculum was developed with the goal of helping students understand trigonometry in terms of an integrated conceptual structure, inspired particularly by the ideas of Robbie Case (Case et al., 1996), and informed by our finding from Part I (Mickey and McClelland, 2025) that grounded rules as well as grounded procedures may contribute to the ability to evaluate trigonometric relationships. We also designed our curriculum in accordance with principles supported by research in cognitive and education science. We included prefatory materials encouraging students to treat mathematics, not just as an exercise in memorizing formulas and procedures, but as an opportunity to understand meaningful relationships. We sought to ensure a solid footing in understanding the component conceptual structures and their relations to symbolic representations before proceeding to integrate them, and we adhered to the education science principle of progressive formalization (Nathan, 2012). Our curriculum was successful in producing robust learning in about 2/3 of the participants included in our controlled comparison study, as measured by performance that exceeded the range of baseline comparisons and remained at about the same level more than 2 weeks after completing the materials. While this outcome fell short of our goal of achieving a 75% success rate, it came close. The pattern of robust learning and retention by about 2/3 of our participants was observed with participants who were not enrolled in a mathematics course, who had no classroom instruction and no contact with instructors, and whose only reward for success (as opposed to mere completion, for which they received payment of $75 and a completion certificate regardless of performance) was a certificate of mastery or excellence based on their post-lesson test score. It is possible, therefore, that even higher levels of success would be observed if the materials were used by students within a mathematics learning setting with opportunities to interact with supportive instructors and peers.

We see these results as consistent with the view that grounding trigonometry—and by extension, many other branches of mathematics—in a meaningful system of relationships rather than treating the subject as an exercise in learning lists of arbitrary rules can lead to successful learning outcomes for many students. We are, of course, not the first to propose that grounding trigonometry and other aspects of mathematics is important. We have drawn inspiration from the writings and textbooks of several mathematicians (Einstein, 1979; Gelfand and Saul, 2001; Henderson and Taimina, 2005; Needham, 2023; Polya, 1945; Stewart, 1995), a mathematical psychologist (Shepard, 2008), and the insights and findings of psychologists and educators interested in mathematics education (Case et al., 1996; Hegarty and Kozhevnikov, 1999; Mayer, 2024; Nathan, 2012; Nathan et al., 2021; Thompson et al., 1994; Uttal et al., 2013; Wertheimer, 1959).

We hope our work has built on the work of these inspiring predecessors, contributing further to our understanding of why grounding matters in two main ways, which we now discuss in turn.

### 4.1 Evidence of two ways grounding can support understanding

First, our findings provide support for the idea that there are two different ways that grounding might support robust learning and transfer of an ability to evaluate trigonometric identities. One way grounding could help is by providing a procedure for solving trigonometric identity problems by visualizing and comparing the polarities and magnitudes of the *x* and/or *y* coordinates of the points on a unit circle referenced by trigonometric expressions. For example, one can see that cos(30) = cos(−30) by visualizing the *x* coordinates of the positions + 30 and −30 and noticing that they are the same. Alternatively, grounding may help by providing a context in which apparently arbitrary rules seem less arbitrary. In the case of the rule cos(−*θ*) = cos(*θ*), grounding helps a student understand this rule as expressing the idea that, for a rotation of any magnitude from the 0 point on the unit circle, the horizontal component of the point you reach is the same, whether the rotation is in the clockwise or the counter-clockwise direction. Such grounded rules can support transfer from one literal problem to another in some cases: For example, if a participant represents both (*θ* − 180) and (*θ* + 180) as *the position you reach by rotating halfway around the unit circle from the position* *θ*, the symbolic expressions sin(30 − 180) and sin(30 + 180) would map to the same representation, supporting transfer from learning sin(*θ* − 180) = −sin(*θ*) to problems relying on the rule sin(*θ* + 180) = −sin(*θ*), and *vice versa*.

In Part I of this two part-project (Mickey and McClelland, 2025), we found support for the view that participants often rely on grounded rules whose meaning derives from the unit circle. Key evidence for this comes from two findings: participants who received our grounded lesson tend to report increased reliance on both the unit circle and rules; and even when they report reliance only on rules, they typically reference spatial relationships on the unit circle when they describe the rules they use. These findings do not rule out a possible role for comparison of coordinate positions, however. The fact that many participants' performance in our Trig Academy study suffered when they did not have access to an external unit circle visualization tool is consistent with the view that these participants relied on this strategy. We note that these two strategies are not mutually exclusive; some participants may rely more on one strategy than the other; indeed, perhaps the subset of successful Trig Academy students who did not rely on the unit circle tool replied primarily on grounded rules. Furthermore, the strategies could be mutually supportive, and/or they may be relied upon to different extents in different contexts. In Study 3 of Part I, participants relied slightly more on the unit circle when the trigonometric relationship was expressed using a generic abstract variable *θ*, and they relied slightly more on rules in taught than transfer problems, consistent with a degree of flexible deployment of alternative grounded solution strategies.

We see the concept of grounded rules as important because it runs counter to the view that visuospatial and formal reasoning are alternative forms of mathematical understanding and is consistent with the idea that understanding mathematical ideas depends on combining visuospatial and symbolic representations in an integrated conceptual structure. Instead of seeing rules strictly as devices for manipulating expressions according to structure sensitive rules, they become expressions of relationships among entities that have meaning in the grounding representation. The unit circle is such a representation: the inferences it supports depend on the ideal object - the perfect circle - rather than an actual specific physical object or the highly schematic rendition of such an object we might try to draw on a marker board or even conjure in space by rotating our forearm around our elbow, as we and some of our participants have done in explaining, for example, why cos(*θ* + 180) = −cos(*θ*).

### 4.2 Achieving success through reliance on principles of learning and education

The studies in Part I provided evidence that those who rely on the unit circle perform better than those who do not, but also suggested that many participants fail to do so, and our pilot study with high school seniors in a pre-calculus trigonometry class underscored this point. The goal of our curriculum was to increase the likelihood of successful engagement with it.

The design of our lesson materials relied on several principles of learning and education. We relied heavily on the principle that understanding of mathematical ideas depends on linking visuospatial and symbolic representations within an integrated conceptual structure. The idea that grounding provides both a procedure for comparing the values of sines and cosines of quantities expressed in degrees and a framework for grounding rules about the relationships in a meaningful conceptual framework shaped the multifaceted approach we took in formulating the details of the presentation of the material covered in the lessons. In addition, we were guided by several widely recognized principles of effective didactic practice: grounding understanding in perception and action, encouraging prior mastery of components, requiring active engagement, providing immediate feedback and requiring practice to promote fluency. We also relied on the principle of progressive formalization of mathematical concepts (Nathan, 2012). Our approach worked for about 2/3 of those who completed our controlled comparison Trig Academy study, resulting in improved performance on our trig identities test and retention over a two-week delay.

These findings support our hypothesis that a structured sequence of lessons designed in accordance with all of these stated principles can be effective, at least for some students. Our approach does not single out any one of these principles, and so does not allow us to independently assess their individual roles in promoting successful learning. Indeed, the independent assessment of most of these principles might be very difficult, because many of them seem intrinsically interrelated. For example, grounding in perception and action and active engagement seem intrinsically related, as do encouraging mastery of components and practice to promote fluency. Progressive formalization sits at the nexus of these two clusters of principles and also implicitly relies on building an integrated conceptual system. Thus, is seems likely to us that incorporating each of these principles into the design of our curriculum supported the effectiveness of incorporating others and contributed to the extent of the success we have been able to achieve.

What might be done to achieve a higher level of success than we were able to achieve with our curriculum? Providing students who struggled with the last two lessons of the curriculum with additional practice opportunities might have reinforced their learning to the point where they could have performed better on the post-test. More broadly, using the materials within the context of coursework on trigonometry rather than as a completely stand-alone exercise could increase the success rate. This could help ensure that students had the necessary motivation to succeed, allow concepts to gain further grounding in potentially more personally meaningful real-world settings, and provide opportunities to benefit from engagement with teachers and peers. We should also remain open to the possibility—indeed the likelihood—that different students will achieve success in different ways. We found evidence that some successful students made far more use than others of the externally provided unit circle, suggesting that students my differ in the approaches they rely on to succeed. Better identifying and targeting these differences could contribute to achieving a higher level of overall success.

### 4.3 Educational implications

The educational implications of our work extend beyond the basic trigonometric identities that have been our focus here. Our studies could be extended or adapted to address a range of interesting questions at the intersection of cognitive science and education. It seems promising to consider the possibility that greater emphasis on encouraging mastery of the conceptual structures underlying other aspects of trigonometry and other branches of mathematics will be useful for helping students learn. It will be equally important for future work to focus on the barriers students face in each of these areas and on identifying ways these barriers might be overcome.

In considering these extensions, we should acknowledge that some topics in trigonometry, or higher-level mathematical reasoning more broadly, may be more amenable to reliance on conceptual structures like the mental number line or the unit circle than others. In other domains, the underlying conceptual structures may be even more abstract, and can even involve higher-order relationships between different conceptual structures themselves. Research across a range of mathematical topics is needed for a full understanding of the use and interdependence of different representations. Likewise, different individuals may have different abilities, and certainly have different proclivities, which may influence the success of a grounded lesson.

### 4.4 Conclusion

Across our studies in Parts I and II of our two-part investigations, the unit circle revealed itself to be a strong, coherent conceptual structure, providing a fertile ground for learning and understanding trigonometry. While the unit circle can be used without reference to rules to solve trigonometric identity problems by directly comparing the horizontal and vertical coordinates of points indicated by trigonometric expressions, we have argued that successful students often learn and use grounded rules that capture spatial relationships. This grounded understanding facilitates application and generalization of rules to support successful problem solving. Our curriculum was constructed to address the difficulty many students face in mastering the unit circle and the shortcomings of many textbooks and classroom practices. Building it in accordance with a set of principles from cognitive and educational science, our curriculum succeeded in producing robust learning in about 2/3 of the students who completed it. Even greater success could be possible if students completed the curriculum within the context of a supportive classroom environment. Further research should explore this possibility. The approach can also be extended to other aspects of trigonometry and other branches of mathematics, potentially helping students learn a wide range of mathematical topics more effectively.

## Data Availability

The datasets presented in this study can be found in online repositories. The names of the repository/repositories and accession number(s) can be found below: Unit Circle Trigonometry GitHub Repository https://trigacademy.github.io/grounded-unit-circle-trigonometry/index.html.

## Footnotes

1 Because of a scoring error which we subsequently corrected, a small number of participants whose true SM score was below the screening threshold were gated through to participate and completed the study. The results from these participants have not been analyzed.

2
https://trigacademy.github.io/grounded-unit-circle-trigonometry/index.html

3
https://public.trigacademy.org/

4 The within-subject relationship between circle use and accuracy on block 1 was not statistically reliable *b* = 0.34, 95% CI [−0.63, 1.31], *z* = 0.69, *p* = 0.490. This could reflect that circle use was largely all-or-none – 18 of the 39 participants used the tool on less than 25% of trials and 17 used it on more than 75% of trials, and such participants provide little within-subject variation to allow measuring an effect. Additionally, participants may have been more likely to use the circle tool on problems they were more likely to fail, and this could have masked a positive relationship between tool use and success for some students.
