# Supplementary material for: Grounding mathematics in an integrated conceptual structure, part II: intervention study demonstrating robust learning and retention through a grounded curriculum
Source: Front Psychol. 2026 Jan 2;16:1507674. doi: 10.3389/fpsyg.2025.1507674 (PMC12808465; doi:10.3389/fpsyg.2025.1507674)
Supplement: Supplementary file 1 [file Data_Sheet_1.pdf]

frontiers

Supplementary Materials for:

Grounding Mathematics in a Coherent Conceptual Structure, Part II: Intervention Study Demonstrating Robust Learning and Retention through a Grounded Curriculum

Mickey, K. W., Kreisel, L. W., Su, S., & McClelland, J. L.[1]
Stanford University, Stanford, CA, USA

[1] Corresponding Author, jlmcc@stanford.edu

1 APTITUDE AND MATH SKILLS AND THEIR RELATION TO TRIG ACADEMY LEARNING OUTCOMES

1.1 Aptitude and Math Skills Assessment Test Battery

We created a Attitude, and Math Skills Assessment (AMSA) test battery which consisted of 14 tests assessing aptitudes, basic math proficiency, and trigonometry-specific math proficiency, as well as several questions assessing math attitudes. The purpose of the AMSA was to help us identify ability and/or preparation related factors that may predict how well students will perform on our materials and give us the opportunity to provide additional support to students who may not have the requisite skills needed to succeed on our existing curriculum. Full details of the materials used in these tests is available in our Unit Circle Trigonometry repository (`https://trigacademy.github.io/grounded-unit-circle-trigonometry/index.html`).

Our aptitude tests included two visuospatial, one verbal, and one relational reasoning test. These tests are commonly used measures: **Mental Rotation Test** (Vandenberg and Kuse, 1978): Students were presented with images of two assemblies of 3D blocks rotated to different positions and asked to decide whether the objects were the same or different. **Embedded Figures Test** (Witkin, 1971): Students were shown a simple figure and a larger complex figure and asked to decide whether the simple figure was hidden somewhere within the complex figure. We obtained the next two tests from the factor-referenced tests provided by the Educational Testing Service (Ekstrom et al., 1976): **Vocabulary Test**: Students were presented with a word and asked to find the best synonym for that word among five answer choices. This task allowed us to assess whether differences in vocabulary might be partially responsible for individual differences in performance in our trig lessons, since these lessons rely on complex language to explain concepts. **Letter Sets Test**: Students were presented with five sets of letters with four letters in each set and asked to find which letter set did not fit the pattern of the remaining sets. This task is thought to tap relational reasoning ability associated with general intelligence without regard to processing modality.

We developed a set of math skills assessment tasks to assess prior skills that might be relevant to trigonometry. This included the following tests: Prior Math Add/Subtract, a page that contained one or two-digit addition and subtraction problems; Prior Math Mark XY, test that asked students to mark positions on the XY plane specified as a pair of [x,y] coordinates; Prior Math Mark Theta, a test that asked students to mark angular positions on a circle specified in degrees in the range from -360 and 360; Prior Math Triangle Trig, a test that presented students with right triangles and asked them to identify sides of the triangle and find sine/cosine/tangent values; Prior Math Circle Trig Concepts, a page that presented students with various angles marked on the unit circle and asked students to find the sine and cosine values of the various angles; and Prior Math Trig Relations. This last task presented students with a subset of the trigonometric identities test questions from the post-test, to allow us to assess whether potential participants already understood the trig identities targeted by our lessons (participants who scored above the 67% correct level on these questions were excluded from completing the lessons and the post-test).

We also created a set of supported math tests to determine whether students' ability to perform well on these tasks once the relevant conventions have been made clear might predict their ability to do well on our lesson materials. These tasks included 1) Supported Mark XY, a page that asked students to mark numbers on an XY plane while reminding them of the conventions for representing positions the XY plane; 2) Supported Mark Theta, a page that asked students to mark angles designated in degrees on a circle while reminding them of the conventions for representing circle positions using degree measure; 3) Supported Triangle Trig, a page that asked students to find the sine, cosine, and tangent of angles in triangles while providing the definitions of these relationships; and 4) Supported Circle Trig Concepts, a page that asked students to find the sine and cosine values of various angles marked on the unit circle, while providing

information about conventions that would help them answer the questions. In addition the these Aptitude and Math Skills measures, we obtained students responses to a set of math attitude and spatial proclivity questions and self reports of the prior mathematics course work.

Over the course of several years, a total of 1967 community college students completed the full AMSA test suite and the additional math attitude, spatial proclivity, and prior mathematics questions. The full data set of results from all of these participants in available in our Unit Circle Trigonometry repository (https://trigacademy.github.io/grounded-unit-circle-trigonometry/index.html). In Figure S1 we provide the correlation matrix based on the full set of participants' performance, together with the wording of the attitude and aptitude questions.

One observation from this matrix is that the Letter Sets aptitude task is more highly correlated with performance on many of the math tasks than the Embedded Figure and the Figure Rotation aptitude tasks; and the observation that, among the math attitude questions, the "I am good at math" and "I find math more exciting than intimidating" questions are most positively correlated with performance on math tasks, and the other attitude questions have very weak positive correlations.

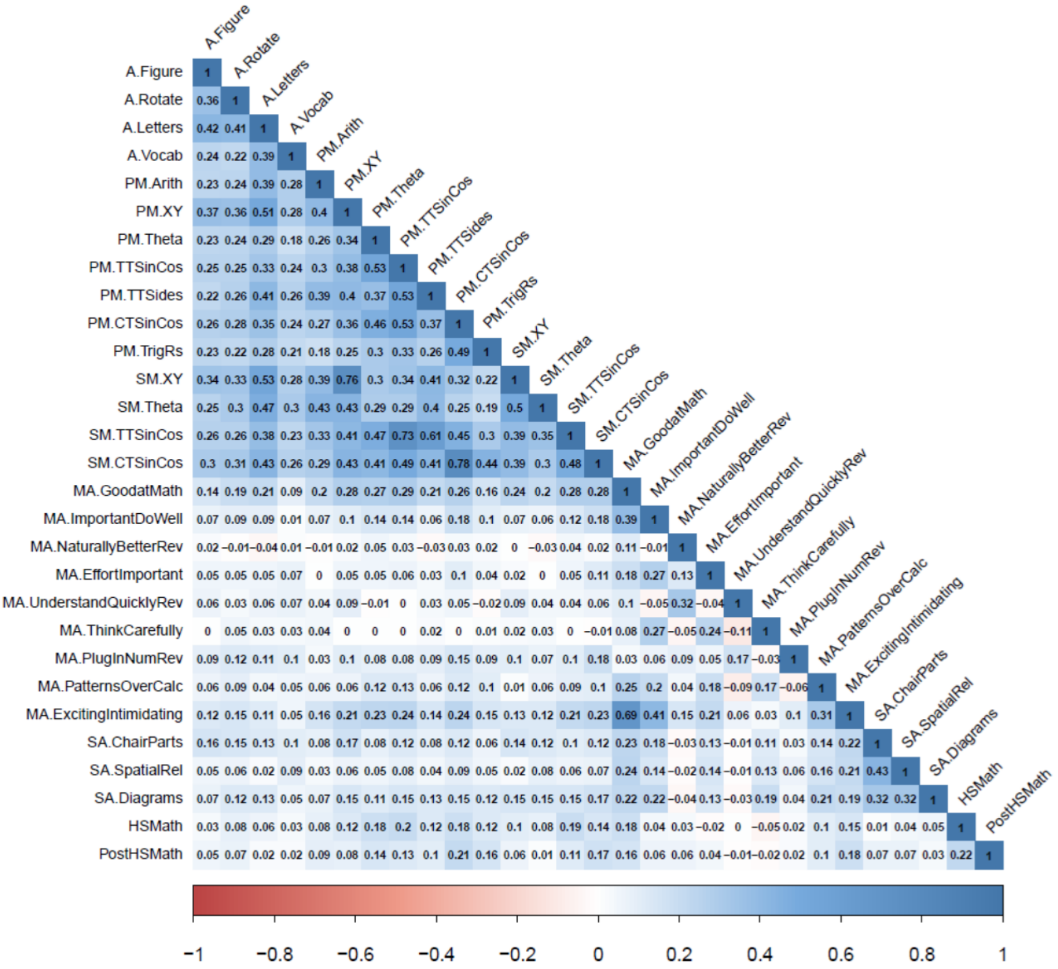

Figure S1. Correlation matrix showing correlations of AMSA test Aptitude (A), Prior Math (PM), Prior Supported Math (SM), Math Attitude (MA), and Spatial Ability (SA) measures with each other. Key: A.Figure=AMSA Aptitude Embedded Figure task; A.Rotate=AMSA Aptitude Figure Rotation task; A.Letters=AMSA Letter Sets task; A.Vocab=AMSA Vocabulary task; PM.Arith=AMSA Basic Math – Arithmetic task; PM.XY=AMSA Basic Math – Mark XY task; PM.Theta=AMSA Basic Math – Mark Theta task; PM.TTSinCos=AMSA Trig-Specific Math task – Find Sin Cos Values on Triangle; PM.TTSides=AMSA Trig-Specific Math task – Identify Sides of Triangle; PM.CTSinCos=AMSA Trig-Specific Math task – Find Sin Cos Values on Unit Circle; PM.TrigRs=AMSA Trig-Specific Math task – Trig Identities task; SM.XY=AMSA Supported Math task – Mark XY; SM.Theta=AMSA Supported Math task – Mark Theta; SM.TTSinCos=AMSA Supported Math task – Triangle Trig; SM.CTSinCos=AMSA Supported Math task – Unit Circle Trig; MA.GoodatMath=I am good at math; MA.ImportantDoWell=It is important to me that I do well in math; MA.NaturallyBetterRev=Some people are naturally better at math than others (reverse coded); MA.EffortImportant=The effort we put into learning math is more important than our innate abilities; MA.UnderstandQuicklyRev=People who are good at math are those who understand new math ideas really quickly (reverse coded); MA.ThinkCarefully=To do well in math it's important to take your time and think carefully; MA.PlugInNumRev=When I do math, I look for formulas I can plug numbers into without thinking too much about the specific context or meaning of the problem (reverse coded); MA.PatternsOverCalc=Math is a subject of patterns that describe the world more than a subject of calculations and rules; MA.ExcitingIntimidating=I see math more as "exciting" than "intimidating"; SA.ChairParts=I would be good at seeing how several parts should be put together to make a piece of furniture like a chair; SA.SpatialRel=I am good at understanding statements that describe spatial relationships; SA.Diagrams=I find diagrams helpful when I try to understand mathematical relationships; HSMath=How many math classes did you take in high school?; PostHSMath=How many math classes have you taken since high school?

1.2 Principal Components and Factor Analysis of Attitude, Aptitude, and Math Skills Measures

We performed a principal component analysis to reduce this data set to a smaller number of components that may be used to later predict performance on our lesson materials and posttest. Figure S2 shows a plot of the first two components that resulted from this analysis. As the plot shows, the first factor (represented with a red arrow, accounting for 22.9% of the variance in the data) contains many of the aptitude and math skills questions, while the second factor (represented with a green arrow, accounting for 8.6% of the variance) contains many of the attitude and spatial proclivity questions.

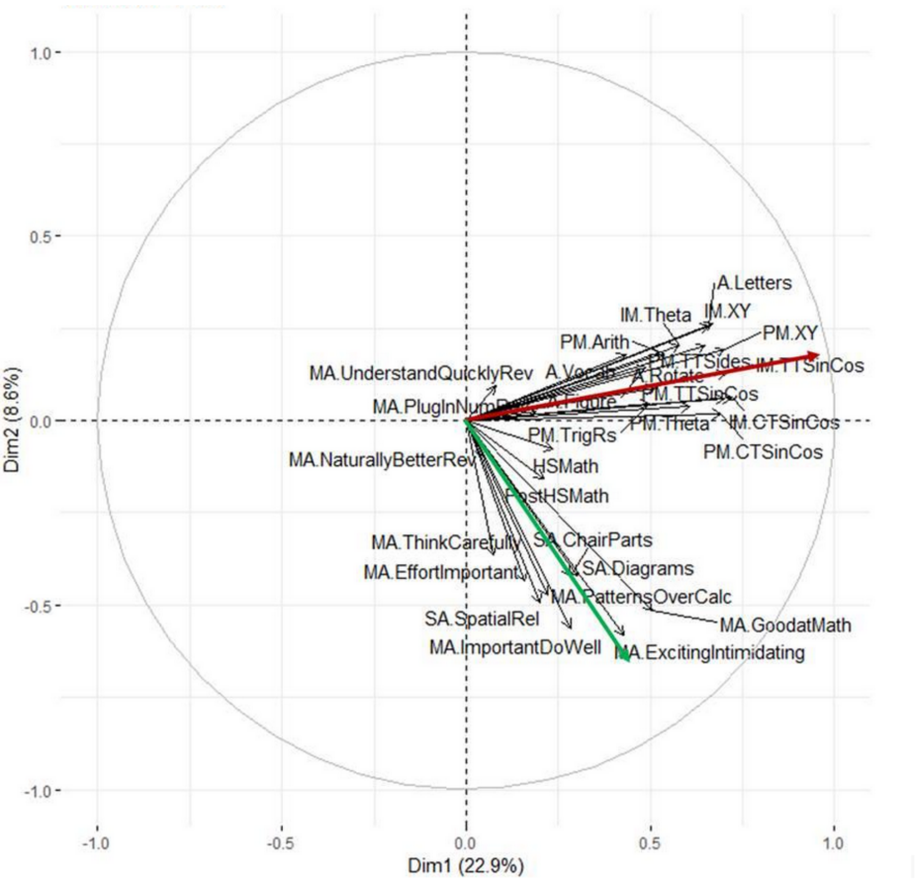

Figure S2. Results of factor analysis of the full set of AMSA tests together with the math attitude and prior coursework questions. The factor analysis results are projected onto the first two dimensions of the principal components analysis of the test scores. See caption to previous figure for details about the particular test elements (in this plot, the label IM corresponds to SM in the previous figure). The first factor, which loads heavily on the many of the aptitude measures is indicated by the red arrow; the second factor, which loads more heavily on math attitudes and spatial abilities, is represented in green.

1.3 Aptitude and Math Skills Predictors of Trig Academy Lesson and Post-Test Performance

We combined results for 62 students (25 high school students from fall 2016, 14 community college students from fall 2016, and 23 high school students from summer and fall 2017) who completed the final

version of our Trig Academy materials (AMSA, lesson, and post-test) prior to conducting the Controlled Comparison Study reported in the main text, to better understand what factors assessed in the AMSA covary with performance on our lesson materials and post-test, and how performance on the post-test covaries with performance on the lesson materials. Figure S3 contains the correlation matrix among the measures used in these analyses in this population of participants.

The matrix shows a high correlation between the last two chapters of materials and performance on the trig identity portion of the trig identities post-test (cell boxed in red, ($r = 0.66, p < 0.001$). Performance on the first four chapters correlated highly with performance on the last two chapters ($r = .66, P < .001$)), but did not correlate as well with performance on the post-test ($r = 0.46, p < .001$).

Among the tasks in the AMSA, the most correlated with performance on the trig identity post-test were the Prior Trig Concepts tasks (cell boxed in green, $r = 0.52, p < 0.001$) which tested students understanding of the the sine and cosine of an angle as defined in triangles, two of the three supported math tasks (Supported Trig Concepts: $r = 0.35, p = 0.005$; Supported Mark Theta: $r = 0.36, p = 0.003$), and the Figure Rotation task ($r = 0.34, p = 0.006$). The supported math tasks were also correlated with performance on the last two chapters of our lesson materials (Supported Trig Concepts: $r = 0.45, p < 0.001$; Supported Mark XY: $r = 0.39, p = 0.002$; Supported Mark Theta: $r = 0.45, p < 0.001$), suggesting that supported math tasks might be most predictive of students' ability to succeed on our curriculum.

A simple linear regression was calculated to predict trig identity posttest results based on student performance on students combined score on the AMSA Supported Math tests. Student performance as measured by this combined score explained a significant proportion of variance in posttest results, $R^2 = 0.20, F(1, 60) = 14.84, p < 0.001$.

A similar linear regression was calculated to predict trig identity posttest results based on student performance on three of the AMSA Aptitude tests (Figure Rotation, Embedded Figure, Letter Sets). Student performance on the combined Aptitude test score also explained a significant proportion of variance in posttest results, $R^2 = 0.16, F(1, 60) = 11.56, p = 0.001$.

We then used multiple regression to further consider these two types of predictors. This analysis showed that the combined Supported Math test score was predictive of performance on the trig identity posttest above performance on the combined Aptitude test score, β=0.42, t(59)=2.05, p=0.045. The combined Aptitude score was not predictive of performance on posttest above the combined Supported Math test score. The two predictors together explained 21.7% of the variance in performance on trig identity posttest $R^2 = 0.22, F(1, 59), p < 0.001$).

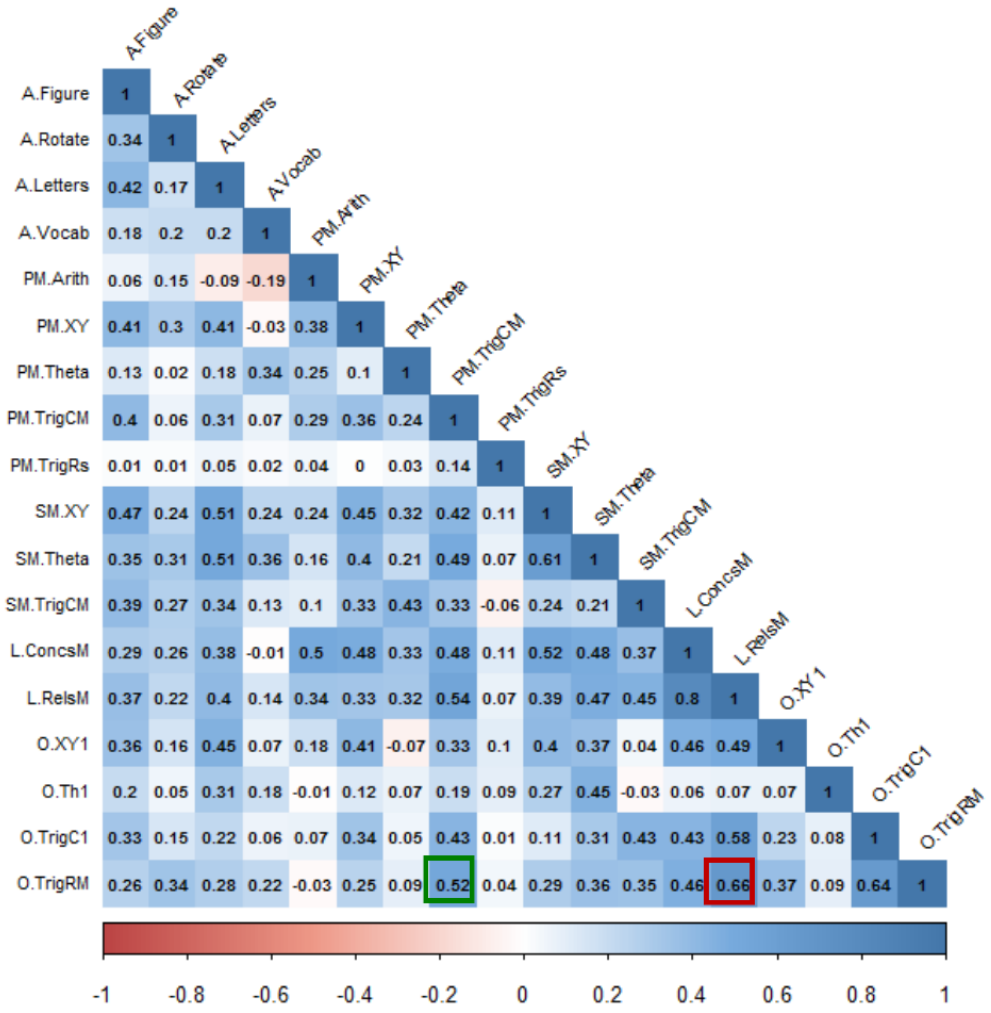

Figure S3. Correlation matrix showing correlations of AMSA test Aptitude (A), Prior Math (PM), and Prior Supported Math (SM) measures with each other and with in-lesson performance (L) as well as Unit 1 outcome (O) measures. Highlighted cells are discussed more fully in the text. Key: A.Figure=AMSA Aptitude Embedded Figure task; A.Rotate=AMSA Aptitude Figure Rotation task; A.Letters=AMSA Letter Sets task; A.Vocab=AMSA Vocabulary task; PM.Arith=AMSA Basic Math – Arithmetic task; PM.XY=AMSA Basic Math – Mark XY task; PM.Theta=AMSA Basic Math – Mark Theta task; PM.TrigCM=AMSA Trig-Specific Math task – Triangle Trig Concepts (corresponds to PM.TTSinCos in Figure S1); PM.TrigRs=AMSA Trig-Specific Math task – Trig Identities task; SM.XY=AMSA Supported Math task – Mark XY; SM.Theta=AMSA Supported Math task – Mark Theta; SM.TrigCM=AMSA Supported Math task – Triangle Trig Concepts (corresponds to SM.TTSinCos in Figure S1); L.ConcsM=Chapter 1-4 of Unit-Circle Curriculum (Concepts); L.RelsM=Chapter 5-6 of Unit-Circle Curriculum (Relations); O.XY1=Mark XY Posttest; O.Th1=Mark Theta Posttest; O.TrigC1=Trig Concepts Posttest; O.TrigRM=Trig Identities Posttest

2 DETAILS OF PREREGISTERED ANALYSES FOR THE TRIG ACADEMY STUDY

The following subsections present the pre-registered analyses that we conducted to examine the effect of the Trig Academy lessons on performance on our 40-item trig identities test, which was administered twice to the participants in both the Lesson Early and the Lesson Late Groups.

These analyses all follow a common approach used to ensure robustness of the findings of these analyses. We view the items used in our test as a fixed and exhaustive set of trig identity item types, and we wished to perform analyses to determine whether the effects of interest, and their possible interaction with the participant's Supported Math (SM) score, to generalize to other participants drawn from the same population. Thus one approach would be to conduct our analyses excluding consideration of problem type altogether and focusing only on overall performance on the full set of items. However, this approach has the potential danger of failing to remove sources of variability due to problem type that would, if removed, allow for greater precision of measurement of the effects of interest. We considered treating problem type as a fixed effect. However, the number of problem types and their interactions make treating problem type as a fixed effect unfeasible (with different means for each problem type). We therefore conducted the analysis twice, once without consideration of problem type, and once with problem type as a random factor. A consistent effect regardless of the inclusion of problem type as a random factor would indicate robustness of the effect. We also assessed statistical reliability with both the Wald test and a bootstrap test.

2.1 Between group effect of lesson

Table S1 presents the logistic regression results for the comparison of the Lesson Early vs. Lesson Late group on the first trig identities test. The Lesson Early group received this test after completing the lessons, which the Lesson Late group waited.

2.2 Within group effect of lesson

Table S2 presents the logistic regression results for the the performance of the participants in the Lesson Late group on the first and second presentations of the trig identities test. The participants in this group completed the lessons between these to presentations of the test.

2.3 Within group effect of delay

Table S3 presents the logistic regression results for the the performance of the participants in the Lesson Early group on the first and second presentations of the trig identities test. The participants in this group completed the lessons before the first test, and then waited before completing the second test, allowing this comparison to determine if there was an effect of the delay between the first and second presentations of the test.

REFERENCES

Ekstrom, R. B., French, J. W., Harman, H. H., and Dermen, D. (1976). Manual for kit of factor-referenced tests. *Princeton, NJ: Educational Testing Service* 586, 1989–1995

Vandenberg, S. G. and Kuse, A. R. (1978). Mental rotations, a group test of three-dimensional spatial visualization. *Perceptual and motor skills* 47, 599–604

Witkin, H. (1971). Group embedded figures test. *APA PsycTests*

Table S1. Results on the first trigonometric identities test for both groups.

	Without problem type			With problem type		
Predictor	*b*	Wald *p*	Boot *p*	*b*	Wald *p*	Boot *p*
(Intercept)	-0.16	.380	.411	-0.16	.489	.524
SM score	0.01	.960	.964	0.00	.986	.988
Group (Lesson Early vs Late)	-0.87	$< .001$	$< .001$	-0.96	$< .001$	$< .001$
Interaction of SM and group	-0.10	.579	.598	-0.17	.385	.427

Table S2. Results within the lesson-late group.

	Without problem type			With problem type		
Predictor	*b*	Wald *p*	Boot *p*	*b*	Wald *p*	Boot *p*
(Intercept)	-0.14	.400	.430	-0.16	.485	.507
SM score	0.08	.654	.689	0.06	.781	.799
Time (TI1 vs TI2)	0.95	$< .001$	$< .001$	1.07	$< .001$	$< .001$
Interaction of SM and time	0.23	$< .001$	$< .001$	0.32	$< .001$	$< .001$

Table S3. Results within the lesson-early group.

	Without problem type			With problem type		
Predictor	*b*	Wald *p*	Boot *p*	*b*	Wald *p*	Boot *p*
(Intercept)	0.68	.018	.036	0.74	.027	.043
SM score	0.05	.868	.882	0.09	.772	.789
Time (TI1 vs TI2)	0.02	.717	.720	0.01	.857	.868
Interaction of SM and time	-0.09	.130	.137	-0.12	.084	.068
